# Maternal protein restriction programs the ageing kidney: Proteomic signatures of early‐life origin renal and metabolic dysfunction

**DOI:** 10.1113/JP291758

**Published:** 2026-08-31

**Authors:** Marina Pereira Pires, Matheus Naia Fioretto, Marcel Rodrigues Ferreira, Isabelle Tenori Ribeiro, Luísa Annibal Barata, Flávia Alessandra Maciel, Lucas Sobrinho Lemos, Renato Mattos, Luiz Marcos Frediane Portela, Patrick Vieira de Souza, Luisa Correia Gomes, Gustavo Monezzi Cordeiro, Wellerson Rodrigo Scarano, Patrícia Aline Boer, Jose Antonio Rocha Gontijo, Luis Antonio Justulin

**Affiliations:** ^1^ Department of Cellular and Molecular Biology, Institute of Biosciences Sao Paulo State University Botucatu São Paulo Brazil; ^2^ Molecular Genetics and Bioinformatics Laboratory, Experimental Research Unit – Unipex, School of Medicine São Paulo State University – Unesp Botucatu São Paulo Brazil; ^3^ Brazilian National Institute of Science and Technology in DOHaD Brazil; ^4^ Fetal Programming and Hydroelectrolyte Metabolism Laboratory, Nucleus of Medicine and Experimental Surgery, Department of Internal Medicine, FCM Campinas State University (UNICAMP) Campinas São Paulo Brazil

**Keywords:** ageing, biomarkers, DOHaD, kidney, maternal malnutrition, proteomics

## Abstract

**Abstract:**

The Developmental Origins of Health and Disease framework proposes that adverse exposures during critical developmental windows induce epigenetic programming, increasing lifelong risk of metabolic and organ dysfunction. Maternal protein restriction (MPR) is a well‐established model of early‐life nutritional stress, associated with reduced nephron number early in life, in addition to renal dysfunction and hypertension during adult life. However, its long‐term impact on renal ageing remains unclear. This study investigated the effects of gestational and lactational severe MPR (6% compared to 17% in controls) on the renal proteome of aged male Sprague–Dawley rats (postnatal day 540) using integrated histological, molecular and *in silico* approaches. MPR induced persistent renal impairment, evidenced by increased serum creatinine and tubular congestion. Proteomic analysis revealed upregulation of proteins related to small‐molecule transport, stress response and energy metabolism, particularly within mitochondrial and redox pathways. By contrast, downregulated pathways involved cytoskeletal organization, vesicular transport, immune processes and post‐transcriptional regulation, suggesting disruption of endomembrane dynamics. Pathway enrichment highlighted central metabolic networks, including glycolysis, pyruvate metabolism and the tricarboxylic acid cycle. Network and microRNA analyses indicated epigenetic regulation, whereas disease enrichment linked findings to metabolic and renal disorders. Nephron‐segment mapping revealed region‐specific alterations. Overall, MPR induces persistent renal programming, disrupting metabolism and function and increasing susceptibility to renal and metabolic diseases with ageing.

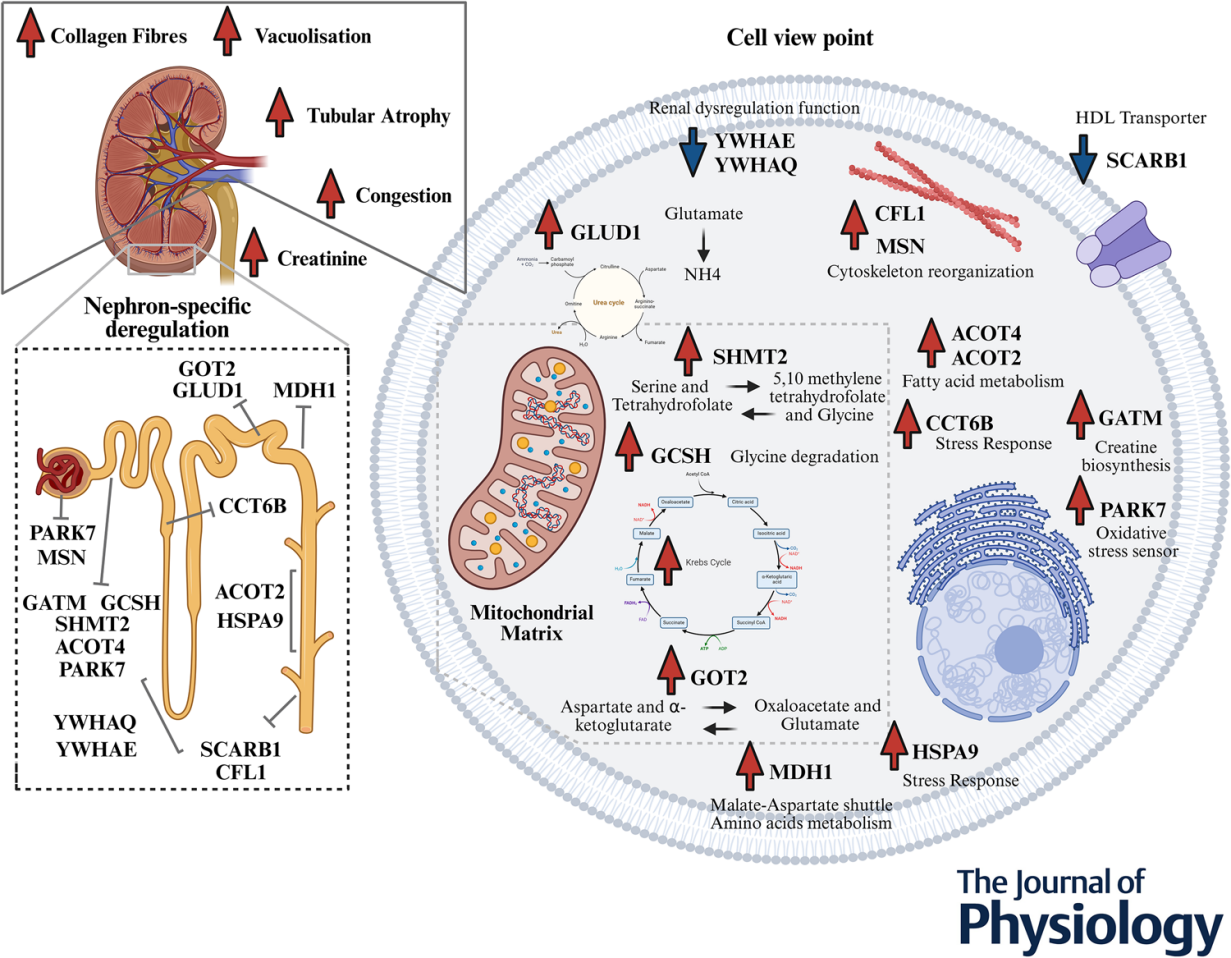

**Key points:**

- Maternal protein restriction (MPR) shifts renal proteome to mitochondrial/redox metabolism.
- MPR causes renal histopathological lesions during ageing.
- The impacts of MPR are reflected in cellular and molecular alterations indicative of potential renal dysfunction.
- Nephron‐specific and epigenetic alterations indicate targeted functional disruption.
- Persistent changes link early malnutrition to renal and metabolic disease risk.

## Graphical abstract

**Abstract figure legend** Maternal protein restriction (MPR) induced persistent renal histopathological alterations accompanied by elevated serum creatinine levels. Nephron‐segment proteomic analysis revealed region‐specific molecular dysregulation affecting Bowman's capsule (PARK7, oxidative stress sensor; MSN, cytoskeletal organization), the proximal convoluted tubule (GATM, creatine biosynthesis; SHMT2 and GCSH, amino acid metabolism; PARK7 and ACOT4, fatty acid metabolism), the loop of Henle (CCT6B, YWHAQ and YWHAE, renal dysfunction), the distal convoluted tubule (GOT2 and GLUD1, amino acid transamination; MDH1, energy metabolism), and the collecting duct (ACOT2, fatty acid metabolism; HSPA9, cellular stress response; SCARB1, HDL transport; CFL1, cytoskeletal organization). The schematic summarizes the macroscopic, nephron‐specific, and cellular mechanisms underlying age‐related renal dysfunction following gestational and lactational MPR. Red arrows indicate upregulated proteins, whereas blue arrows indicate downregulated proteins.

## Introduction

In the 1980s, David Barker investigated the effects of maternal malnutrition during pregnancy on the offspring's health. His research demonstrated that low birth weight, defined as less than 2500 g at birth, increases the risk of cardiovascular disease later in life (Barker et al., 1989). Subsequent studies established the concept of the Developmental Origins of Health and Disease (DOHaD), which links the perinatal environment (i.e. the period immediately before and after birth) to embryonic and fetal development, encompassing the processes of growth and differentiation from fertilization to birth. These developmental stages are critical for growth and are highly susceptible to adverse influences. Consequently, unfavourable conditions during this period can result in lifelong health consequences (Barker, 2004; Barker et al., 1993; Itoh et al., 2022; Ravelli et al., 1976; Tu'akoi et al., 2020).

Maternal protein restriction (MPR) is widely used to study how early‐life conditions affect long‐term health. MPR involves reducing protein intake during pregnancy. This increases the probability of offspring developing metabolic disorders, such as diabetes, and is linked with cardiovascular, renal, behavioural and psychiatric conditions, such as increased incidence of prostate cancer and activation of oncological pathways, a lower number of β cells and decreased insulin production, as well as hypertension and renal and cardiac alterations (Assalin, Gontijo, Boer, 2019; Morimoto et al., 2012; Santos et al., 2019; Su et al., 2016). A key mechanism within this field is the concept of match and mismatch between the environment experienced by a fetus/infant and the environment they encounter as adults (Hanson & Gluckman, 2014).

Gestational MPR is associated with low birth weight, a 28% reduction in nephron number, and impaired renal salt excretion early in life, in addition to a higher risk of chronic kidney failure and hypertension in adulthood (Mesquita et al., 2010; Schreuder et al., 2006; Sene et al., 2013, 2018, 2021). When MPR occurred during pregnancy and lactation, the number of nephrons was reduced by 38%, and blood pressure was higher from week 17 onwards (Lamana et al., 2021). MPR harms kidney development by altering nephrogenesis and disrupting nephron formation. It also affects the synthesis of structural proteins and promotes tissue remodeling, thereby altering kidney tissue architecture. MPR reduces proliferation and promotes early differentiation of renal progenitor cells (Guo et al., 2020; Harrison et al., 2009). These kidney‐specific changes are strongly linked to chronic kidney disease (CKD) and adult hypertension. CKD remains a major global health concern, causing high morbidity and mortality in both Brazil and globally (Couser et al., 2011; Zarocostas, 2010). The World Health Organization estimates CKD affects ∼10% of the global population, and its prevalence is rising. However, the long‐term effects of MPR on kidney function during ageing are unclear. It is not known whether renal histopathological and molecular abnormalities appear during ageing (Cockwell & Fisher, 2020; Eisenstein, 2023; The ISN, 2025).

In this study, we evaluate how MPR affects the renal proteomic profile in ageing male rats at postnatal day (PND)540. This time point is critical for ageing research. We use histopathological, molecular, and *in silico* methods. The central hypothesis is that MPR causes significant metabolic and cellular stress during ageing. This may lead to renal overload and a greater risk of kidney disease compared to controls receiving normal protein.

## Methods

### Animals and experimental design

The experimental procedures followed the National Council for the Control of Animal Experimentation (CONCEA), a Brazilian regulatory agency for animal research, and study approval was obtained from the Ethics Committee on the Use of Animals of the Institute of Biosciences of Botucatu (CEUA n° 5119280121).

Male and female Sprague–Dawley rats were obtained from the Biotherapy Center of the State University of Campinas (Campinas, SP, Brazil). Animals and offspring were housed at the Animal Facility, Department of Morphology, in autoclaved polyethylene cages (two per cage) with wood shavings as substrate. Environmental conditions comprised a 12:12 h light/dark photocycle at 22–25°C. Filtered water and standard laboratory chow were provided *ad libitum*. After confirming pregnancy, 40 rats were individually caged and assigned to either a control (CTR, *n* = 20; 17% protein) or a low‐protein diet (GLLP, *n* = 20; 6% protein; Reeves et al., 1993) during gestation and lactation. The normoprotein diet contained 627.5 g kg^−1^ carbohydrates and 70 g kg^−1^ lipids, whereas the low‐protein diet contained 760 g kg^−1^ carbohydrates and 70 g kg^−1^ lipids. Both diets were isocaloric (3.96 kcal g^−1^), with the lower protein content compensated by increased carbohydrate levels. The nutritional information is provided in Table 1.

**Table 1:** Nutritional information about the diets

Ingredients	Normoprotein (17% protein)	Low‐protein (6% protein)
Starch	397	480
Casein (84% protein)	202	71.5
Dextrin	130.5	159
Sucrose	100	121
Soy oil	70	70
Fibre of pH 101 or pH 102 (microcellulose)	50	50
Mixture of salts AIN93G°	35	35
Mixture of vitamins AIN93G°	10	10
l‐Cystein	3	1
Choline bitartrate (41.1% choline)	2.5	2.5
Energy intake (kcal g^−1^)	3.96	3.96

The low‐protein diet was formulated by Pragsoluções (PragSoluções, São Paulo, Brazil). All diets were supplemented with l‐cystine as the sulphur‐containing amino acid. Vitamin and mineral compositions were adjusted in accordance with the American Institute of Nutrition (AIN‐93G) recommendations for rodent diets. To match the phosphorus content of the control casein diet (3 g kg^−1^), monobasic potassium phosphate was added to the mineral mix, ensuring a consistent calcium‐to‐phosphorus ratio of 1.3 across both diets.

with a significance threshold of *P* < 0.05, based on the binary distance of the dendrograms.

Sample size accounted for potential GLLP losses. Some organs were allocated to other studies for animal use efficiency. At birth, litters were standardized to eight pups per dam (1:1 male: female) to control for maternal behavioural bias. Litters with fewer than eight pups or that did not meet the sex ratio were excluded.

After weaning, animals from both the CTR and GLLP groups received a normoprotein diet until PND540. The females underwent the same treatment and, after weaning, were assigned to other colleagues for reproductive analyses. Here, we used only the male offspring, as noted in the study limitations. At PND540, male offspring were euthanized by intraperitoneal administration of ketamine (75 mg/kg) and xylazine (5 mg/kg), followed by decapitation. [Correction added on 16 September 2026, after first online publication: The route of administration and dosages of ketamine and xylazine have been included.] The experimental design followed the protocol described by Fioretto et al. (2025). After decapitation, blood was collected and centrifuged, and the resulting serum was used for biochemical analysis. Kidneys were extracted and weighed before fixation and histological processing (*n* = 5 per group). Additional kidneys were immediately frozen in liquid nitrogen and stored at −80°C (*n* = 5 per group).

### Biochemical analyses

Blood was collected and plasma (*n* = 6 per group) obtained by centrifugation (4000 rpm/1.699 *g* for 20 min at 4°C). Serum alanine aminotransferase (ALT), aspartate aminotransferase (AST), creatinine, urea, and alkaline phosphatase were measured by calorimetry using the VIDA S1200 system (Vida Biotecnologia, Belo Horizonte, Brazil) in accordance with the manufacturer's instructions.

### Histological analyses

Methods followed by Sobrinho Lemos et al. (2026). Kidneys (*n* = 5 per group) were fixed in methacarn (60% methanol, 30% chloroform, and 10% acetic acid) for 4 h. They were then dehydrated in graded ethanol, cleared in xylene, and embedded in Paraplast (Sigma‐Aldrich, St Louis, MO, USA). Five‐micron‐thick sections were cut with a rotary microtome, mounted on silanized slides and stored for staining.

For morphological evaluation, sections were stained with haematoxylin and eosin and analyzed using a DM2500 light microscope equipped with a digital camera and Qwin V3 software (Leica, Wetzlar, Germany). Histopathological assessment (*n* = 5 per group) was performed in a blinded manner using a semi‐quantitative scoring system: 0 (absent), 1 (0–15%), 2 (16–30%), 3 (31–60%) and 4 (61%–100%), evaluating cortical (outer kidney layer) and medullary (inner kidney region) congestion (dilatation of vessels associated with the glomerulus and/or peritubular veins and capillaries), tubular atrophy, vacuolization, interstitial fibrosis and inflammatory foci.

Mast cells were identified by toluidine blue staining (a dye that specifically highlights mast cell granules). Quantification was performed in 10 random fields per animal (40× magnification; *n* = 5 per group), totaling 60 fields per group, following an adapted protocol.

Periodic acid–Schiff (PAS) staining detected glycogen and glycoproteins. Ten random images per animal (40×; *n* = 5 per group; 60 images per group) were analyzed, and the results were reported as stained area percentage using ImageJ (NIH, Bethesda, MD, USA). Collagen, reticular and elastic fibres were assessed using picrosirius red, reticulin or fuchsin. Quantification used automatic detection in 10 fields per animal (40×; *n* = 5 per group), with results reported as fibre area proportion of the total field.

The microscopic field refers to the field of view observed under the microscope and is shown as a frame captured with ImageJ, version 1.45 (NIH). Data were measured in length using a photomicrograph scale (µm). We quantified the area occupied by the specimen (the portion of the image filled by the specimen) relative to the total frame area for each photomicrograph. Analyses were performed using a DMLB 80 microscope (Leica), which was connected to a DC300FX digital camera (Leica) and image analysis software (Leica).

### Global protein expression by liquid chromatography‐tandem mass spectrometry (LC‐MS/MS)

#### Protein extraction, quantification and digestion

Kidney samples (100 mg; *n* = 5 per group) were homogenized on ice for 5 minutes in 1000 µL of extraction buffer (comprised of 8 m urea, a strong protein denaturant; 100 µL of 1 m Tris, a buffering agent; phenylmethanesulfonyl fluoride (PMSF), a phosphatase inhibitor; 1% protease inhibitor, which prevents protein degradation; and 65 mm DTT). The homogenate was ultrasonicated with ultrapure water at 4°C for 5 min, then centrifuged at 4°C for 30 min at 14,000 rpm/20.817 *g*. This was repeated three times. The supernatant was collected, and total protein was quantified using the Bradford Test (Bradford, 1976). The Quick Start Bradford Protein Assay kit (Bio‐Rad, Hercules, CA, USA) was used in duplicate.

#### Acquisition of nanoLC‐electrospray ionization‐MS/MS data for proteomic analysis

Peptide analyses were carried out on an Xevo G3 Q‐TOF mass spectrometer, coupled to an ACQUITY UPLC M‐CLASS system (a high‐resolution LC platform for separating peptide mixtures; both from Waters Corp., Milford, MA, USA). The system was controlled by MassLynx, version 4.2 (Waters Corp.). Chromatographic separation was done on a reversed‐phase nanoEASE M/Z HSS T3 analytical column (75 µm × 150 mm, 1.8 µm; Waters Corp.), which was pre‐equilibrated with 7% acetonitrile containing 0.1% formic acid. Peptides were separated using a linear gradient from 7% to 40% of mobile phase B (mobile phase B is 0.1% formic acid in acetonitrile) over 70 min at a flow rate of 0.35 µL min^−1^. The column temperature was maintained at 40°C.

The mass spectrometer was operated in positive electrospray ionization mode under data independent acquisition (MSE). Precursor ions were continuously scanned over the range *m*/*z* 50–2000 in the quadrupole (Q) analyzer. These were then transmitted to the time‐of‐flight (TOF) analyzer during the low‐energy cycle for full‐mass detection at 40,000 full‐width at half‐maximum resolving power. In the following high‐energy cycle, ions underwent collision‐induced dissociation in the transfer cell. Fragment ions were then analyzed in the TOF within the same *m*/*z* range. Source parameters were: capillary voltage 2.8 kV; sampling cone 30 V; extraction cone 5 V; source temperature 80°C. Data were acquired over 70 min, covering the mass range 50–2000 Da. A lockspray system ensured mass accuracy and reproducibility using a [Glu^1^]‐fibrinopeptide B solution (500 fmol µL^−^^1^) infused at 0.5 µL min^−1^. The reference ion was monitored at *m*/*z* 785.8426 in positive mode. Samples contained enolase as an internal standard at 100 fmol.

#### Protein profile determination

The analyses were conducted according to the methods described by Naia Fioretto et al. (2024) and Fioretto et al. (2025). Results were analyzed with ProteinLynx Global Server (PLGS) software (Waters Co., Manchester, New Hampshire, UK), which is used for mass spectrometry data processing, to identify *Rattus norvegicus* (rat) proteins using the UniProt protein sequence database (https://www.uniprot.org) and statistical Monte Carlo algorithms. For quantitative proteome analysis, only proteins with a confidence score above 95% (indicating the statistical certainty of protein identification) were included. In comparing groups (GLLP *vs*. CTR at PND540), proteins were classified as differentially expressed proteins (DEP). Proteins were considered downregulated when *P* ≤ 0.05 (indicating a statistically significant decrease) or when unique to the CTR group, and upregulated when *P* ≥ 0.95 (indicating a statistically significant increase) or unique to the GLLP group. The cutoffs *P* ≤ 0.05 and *P* ≥ 0.95 were used to increase the reliability and significance of the findings. Raw data from the proteomic analyses are available in the Supporting information (File S1) for further studies.

#### Proteomic analysis statistics

Cellular and biological functions were identified using Gene Ontology (GO) (http://geneontology.org) classification, a system that organizes genes into categories based on known biological roles. Protein analysis was further refined using the Protein Annotation Through Evolutionary Relationships (PANTHER) database (http://pantherdb.org/), which classifies proteins based on their evolutionary history. Protein codes were identified in UniProt, a database of protein sequences and functional information, and converted into gene names using the Gene ID conversion tool (https://www.uniprot.org/).

### Functional annotation analysis and data representation: *in silico* approach

#### Data selection, integration and general enrichment of biological, cellular and molecular parameters

Proteomic analyses of kidneys at PND540 identified 491 DEP, including 303 upregulated and 188 downregulated (see Supporting information, File S1). After statistical exclusion, removal of unfound proteins, and elimination of duplicates, 491 DEP remained.

We accessed KOBAS 3.0 (http://kobas.cbi.pku.edu.cn/) to identify 12 pathways and cellular processes enriched by Kidney DEP, using the data by Kyoto Encyclopedia of Genes and Genomes (KEGG) (https://www.genome.jp/kegg), Reactome, Panther and GO (Bu et al., 2021) (see Supporting information, Files S2 and S3). Bar plots were generated in Prism, version 8.0.1 (GraphPad Software Inc., San Diego, CA, USA), with corrected *P* values converted to –log10.

Ontological enrichment for the 10 most significant molecular processes (GO Molecular Function 2025) (see Supporting information, Files S4 and S5) was performed for upregulated and downregulated DEP using Enrichr (https://maayanlab.cloud/Enrichr/) (Chen et al., 2013; Kuleshov et al., 2016; Xie et al., 2021), considering *P* value significance. Appyter (https://appyters.maayanlab.cloud/#) was used to generate the graphs. Enrichr also examined the 10 most enriched metabolites, integrating with human data (Misc HMDB metabolites) (see Supporting information, Files S6 and S7) based on adjusted *P* values.

#### General data filtering, biochemical analyses and epigenetic potential

From all the enrichment, we performed protein–protein integration for upregulated and downregulated DEP, respectively, in the STRING platform (https://string‐db.org) (Szklarczyk et al., 2015), using multiple proteins for *R. norvegicus*, with the following parameters: Full string network, 0.900 highest confidence, to investigate the major interactions between proteins, in addition to demonstrated the annotated keyword (Uniprot) enrichment, considering false discovery rate (FDR) and gene count. In addition, we used both downregulated and upregulated DEP to visualize the interactions, using ShinyGO 0.85.1 (Ensembl/STRING‐db ID – rnorvegicus_gene_ensembl – *Rattus norvegicus*; Rat genes mRatBN7.2 Taxonomy ID 10116 – Source ENSEMBL) (https://bioinformatics.sdstate.edu) (Ge et al., 2020; Kanehisa et al., 2021; Luo & Brouwer, 2013), using the pathway database KEGG, with FDR cutoff 0.05. The chart was represented by dotplot, representing the fold enrichment, number of genes (DEP), and –log10 (FDR) (see Supporting information, File S8). In addition, we represented three KEGG pathways using the same tool.

After that, we selected only proteins with protein–protein interactions to investigate the epigenetic potential of regulating these DEPs. Subsequently, we investigated the validated and predicted microRNAs (miRNAs) in the MirWalk platform (http://mirwalk.umm.uni‐heidelberg.de) (Sticht et al., 2018), selecting the analysis for rat (see Supporting information, File S9). The interaction graphs were constructed in the SankeyMATIC platform (https://sankeymatic.com). To visualize these interactions (57 DEP), we used ShinyGO 0.85.1 (Ensembl/STRING‐db ID – rnorvegicus_gene_ensembl – *Rattus norvegicus*; Rat genes mRatBN7.2 Taxonomy ID 10116 – Source ENSEMBL) (https://bioinformatics.sdstate.edu) (Ge et al., 2020; Kanehisa et al., 2021; Luo & Brouwer, 2013), using the pathway database Curated.GeneSetDB, and using the Tree graph with pathways with many shared genes are clustered together. Bigger dots indicate more significant *P* values. KEGG was used to confirm the metabolic pathways observed previously, with a FDR cutoff of 0.05. The chart was represented by a dotplot, representing the fold enrichment, number of genes (DEP) and –log10 (FDR) (see Supporting information, File S10). We also included the information about the DEP/genes and chromosome enrichment, using the characteristics of your genes and the rest in the genome. Chi‐squared and Student's *t* tests were applied.

#### Potential risk disease enrichment

To investigate the associations between our genes of interest and human diseases, we performed a functional enrichment analysis using gene sets from the DISEASES database (https://maayanlab.cloud/Harmonizome/resource/DISEASES) of the 57 DEP. This database integrates evidence of associations between genes and diseases from multiple sources, including: (i) disease gene evidence scores by manual literature curation; (ii) disease gene evidence scores by integrating experimental data (genome‐wide association studies, GWAS); and (iii) Gene‐disease co‐occurrence scores from text‐mining biomedical abstracts. The analysis was conducted in the R environment, version 4.3.0 (@Manual) (R Core Team, 2022), using the clusterProfiler (Wu et al., 2021; Yu et al., 2012), GSEABase (Morgan et al., 2021) and tidyverse (Wickham et al., 2019) packages for data processing and analysis. Statistical significance was assessed using the Benjamini–Hochberg method to correct for multiple testing, with a significance threshold of *P* < 0.05. Heatmaps and chord diagrams were created using the ComplexHeatmap (Gu et al., 2016) and the circlize (Gu et al., 2014) packages, respectively. A BibTeX entry for LaTeX users is @Manual{, title = {R: A Language and Environment for Statistical Computing}, author = {{R Core Team}}, organization = {R Foundation for Statistical Computing}, address = {Vienna, Austria}, year = {2022}, url = {https://www.R‐project.org/},}. The circos plot and heat map were constructed based on statistical analysis of binary distance for the dendrograms (see Supporting information, File S11).

#### Enrichment of targets with greater epigenetic interaction and integration

Finally, we selected targets regulated by more than one microRNA (miRNA) (CCT6A, CFL1, CYP4A8, EXOSC2, GATM, GCSH, GLUD1, GOT2, VIM, YWHAQ, HSPA9, ITSN1, MDH1, MSN, OXCT1, PARK7, MYL1, P4HB, RPS27A, SCARB1, YWHAE, ACOT2, ACOT4, AK2, ALDH6A1, ATP1B1, ATP6V1A, SHMT2, TUBA1A). Of these, we used only those that interacted with each other. For this, we used a performed protein–protein integration for upregulated and downregulated DEP, respectively, in the STRING platform (https://string‐db.org) (Szklarczyk et al., 2015), using multiple proteins for *R. norvegicus*, with the following parameters: full string network, 0.700 high confidence, to investigate the major interactions between proteins, in addition to demonstrating the Subcellular Localization (COMPARTMENTS) enrichment, considering FDR and gene count.

Therefore, we used the selected targets associated to clusters in the protein–protein interaction: CFL1, GATM, GCSH, GLUD1, GOT2, YWHAQ, HSPA9, MDH1, MSN, PARK7, SCARB1, YWHAE, ACOT2, ACOT4, SHMT2, CCT6A. To enrich the specific metabolites associated with these DEPs, we used the MetaboAnalyst 6.0 platform (https://www.metaboanalyst.ca) using Network explorer (Human official gene symbol) – Gene‐Metabolite Interaction Network (degree cutoff 2.0; filters nodes based on degree cutoff 2.0) – Database KEGG (G) (Pang et al., 2024). In addition, we used ShinyGO 0.85.1 (Ensembl/STRING‐db ID – rnorvegicus_gene_ensembl – *Rattus norvegicus*; Rat genes mRatBN7.2 Taxonomy ID 10116 – Source ENSEMBL) (https://bioinformatics.sdstate.edu) (Ge et al., 2020; Kanehisa et al., 2021; Luo & Brouwer, 2013), using the pathway database Curated.WikiPathways (FDR cutoff 0.05), considering the number of genes (DEP), –log10 (FDR), and fold enrichment (aspect ratio in 2), and represented by a plot chart (see Supporting information, File S12).

#### Kidney‐nephron‐specific expression of key biomarkers associated with renal disorders

Finally, we accessed the Kidney Cell Explorer platform (https://cello.shinyapps.io/kidneycellexplorer) based on Single‐Cell Profiling Regional Diversity in the Mouse Kidney by RNA sequuencing datasets (Ransick et al., 2019), using normalized log expression values ​​with mean (rescaled) expression to visualize the expression of the 16 DEPs in the nephron and ureteral epithelium, vasculature, immune and interstitial cells, as well as the specific expression of the nephron–ureteral region. The only limitation of this analysis was that the database lacked information on the CCT6A target, and, by biological approximation, we used the CCT6B isoform.

### Statistical analysis

Statistical analyses were performed using Prism, version 8.0.1 (GraphPad Software Inc.). The Shapiro–Wilk test was used to test the normality of the data. The parametric data were submitted to Student's *t* test, and non‐parametric data were evaluated by the Mann–Whitney test. Data were expressed as the mean ± SD or SEM. *P* < 0.05 was considered statistically significant.

## Results

No differences in body or kidney mass were observed between groups (Table 2). Serum analysis showed no changes in AST, ALT, alkaline phosphatase, or urea, but creatinine was higher in the GLLP group compared to the CTR group.

**Table 2:** Biometric and metabolic parameters in the offspring at PND540

Parameters	CTR	GLLP	
Body mass (g)	537.9 ± 126.5	513.3 ± 61.3	*P* = 0.5380
Kidney mass (g)	4.121 ± 0.6	3.726 ± 0.6	*P = 0.1205*
AST (U L^−1^)	102.0 ± 30.1	105.0 ± 61.7	*P = 0.9037*
ALT (U L^−1^)	6.7 ± 1.5	10.0 ± 3.6	*P = 0.0620*
Creatinine (mg dL^−1^)	0.59 ± 0.09	0.76 ± 0.13^*^	*P = 0.0337*
Alkaline phosphatase (U L^−1^)	281.6 ± 77.69	391.0 ± 101.0	*P = 0.0649*
Urea (mg dL^−1^)	22.53 ± 3.672	23.62 ± 1.796	*P = 0.2403*

* Statistically significant difference between control (CTR) and gestational and lactational protein restriction (GLLP). Data are presented as the mean ± SEM (*n* = 6 per group). Statistical analysis by Student's *t* test, with significance when *P* < 0.05 (indicated in italics).

In histopathological analyses, we observed a significant increase in cortical and medullary congestion (Fig. 1*I–I*), tubular atrophy (Fig. 1*I–II*), and areas of vacuolization (Fig. 1*I–V*) in the GLLP kidneys, although there were no differences in inflammatory foci (Fig. 1*I–III*). Furthermore, our results demonstrated a significant decrease in reticular fibres (Fig. 1*II–IV*) and an increase in glycoproteins (Fig. 1*III–VI*) in the GLLP group. Regarding the quantity of mast cells and elastic fibres, there was no difference between the experimental groups (Fig. 1*IV–VII* and *V–VIII*). Finally, we observed an increase in the area of ​​collagen fibres in the GLLP group (Fig. 1*VI–IX*) and an increase in interstitial fibrosis (Fig. 1*VI–X*).

**Figure 1:**
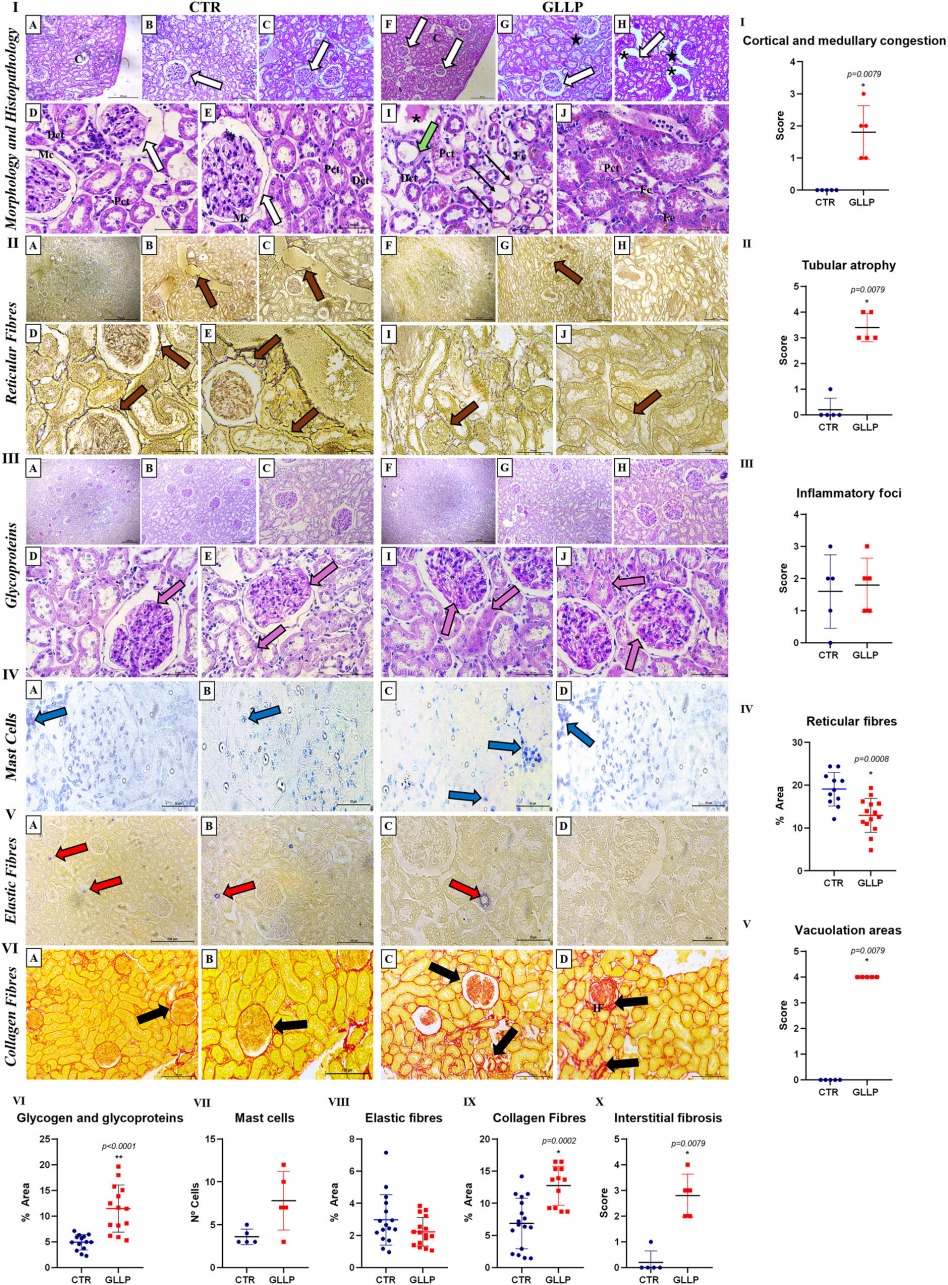
Morphological, morphometric and histopathological analyses of the kidneys of CTR and GLLP animals in PND540 ***I***, haematoxylin and eosin staining. ***A***–***E***, representative images of the kidneys from the CTR group, showing preserved architecture, with well‐defined cortex and medulla (white arrows). ***F***–***J***, images of the kidneys from the GLLP group, showing a significant increase in cortical and medullary congestion (black arrows), tubular atrophy (green arrows) and areas of vacuolization (black and white arrows). Graph ***I***: significant increase in cortical and medullary congestion in the GLLP group. Graph ***II***: significant increase in tubular atrophy in the GLP group. Graph ***III***: non‐significant difference in inflammatory foci between the groups. ***II***, reticulin staining. ***A***–***E***, images of the CTR group, with reticular fibres well distributed around the tubules and glomeruli (brown arrows). ***F***–***J***, images of the GLLP group, demonstrating a significant decrease in these fibres (brown arrows). Graph ***IV***: significant reduction of reticular fibres in the GLLP group. ***III***, glycoprotein analysis by PAS staining. ***A***–***E***, images of the CTR group, with discrete and homogeneous staining. ***F***–***J***, images of the GLLP group, showing an increase in PAS‐positive areas (purple arrows), indicating glycoprotein accumulation in the extracellular matrix. Graph ***V***: increased vacuolization in the GLLP group. Graph ***VI***: significant increase in glycoproteins in the experimental group. ***IV***, toluidine blue staining. ***A*** and ***B***, images of the CTR group, with low mast cell density (blue arrows). ***C*** and ***D***, images of the GLLP group, showing a significant increase in mast cells (blue arrows). Graph ***VII***: quantitative increase in mast cells in the GLLP group. Graph ***VIII***: elastic fibres in the experimental groups. ***V***, fuchsin staining. ***A*** and ***B***, images of the CTR group, with well‐distributed elastic fibres (red arrows). ***C*** and ***D***, images of the GLLP group, demonstrating a significant reduction in these fibres (red arrows). Graph ***IX***: increase in collagen area in the GLLP group. ***VI***, picrosirius staining. ***A*** and ***B***, images of the CTR group, with collagen restricted to normal areas of the interstitium (black arrows). ***C*** and ***D***, images of the GLLP group, showing a marked increase in collagen deposition (black arrows), characterizing renal fibrosis (Graph ***X***). Graph ***IX***: significant increase in the total collagen area in the GLLP group. *n* = 5 per group. The graphs present the mean ± SD, considered significant when *P* < 0.05. *Magnification 4× to 40×.

In the proteomic analyses of kidneys at PND540, we found 491 DEP, 303 of which were upregulated and 188 downregulated. The upregulated DEP enrichment showed the following biological pathways: Metabolic pathways, Carbon metabolism, Glycolysis/Gluconeogenesis, Biosynthesis of amino acids, Metabolism of amino acids and derivatives, The citric acid cycle and respiratory electron transport, Cellular responses to stress, Cysteine and methionine metabolism, 2‐Oxocarboxylic acid metabolism, Peroxisomal protein import, Innate immune system, and Transport of small molecules (Fig. 2*A*); cellular components: Mitochondrion, Cytosol, Oxidation‐reduction process, Identical protein binding, Mitochondrial matrix, Cytoplasm, Hydrogen peroxide catabolic process, Glycolytic process, Tricarboxylic acid cycle, NAD binding, Enzyme binding, and Response to oxidative stress (Fig. 2*B*); molecular parameters: Oxidoreductase activity, acting on the aldehyde or oxo group of donors, NAD or NADP as acceptor, Oxidoreductase activity, acting on the CH‐OH group of donors, NAD or NADP as acceptor, MHC class II protein complex binding, Oxidoreductase activity, acting on the aldehyde or oxo group of donors, Disulfide as acceptor, ADP binding, ATP binding, Adenyl ribonucleotide binding, Disordered domain specific binding, Ubiquitin protein ligase binding, and Ubiquitin‐like protein ligase binding (Fig. 2*C*); and metabolites: NADH, NAD, Oxoglutaric acid, Pyruvic acid, Carbon dioxide, l‐Cysteine, ACO, l‐Glutamic acid, QH2, and Coenzyme Q (Fig. 2*D*).

**Figure 2:**
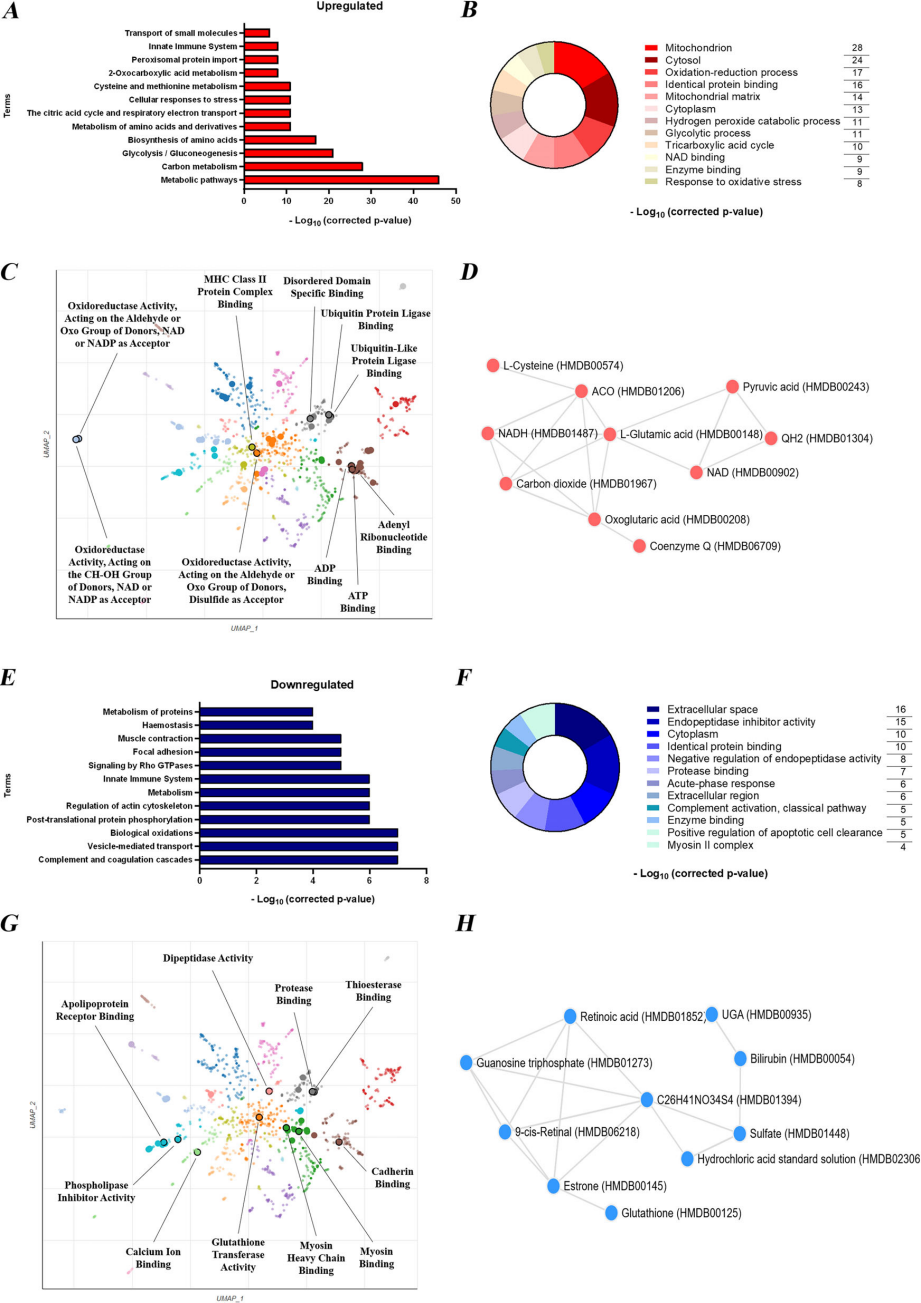
General biological enrichment of the upregulated and downregulated DEP. Enrichment of 12 upregulated (A) and downregulated (E) biological pathways (A and E) elucidating aspects of carbohydrate, energetic, and amino acid metabolism Data were obtained from the KOBAS 3.0 platform, using the Panther, Reactome, GO and KEGG databases. Enrichment of upregulated (***B***) and downregulated (***F***) cellular processes (B and F) associating mainly with structural DEP. Analyses were performed in the KOBAS 3.0 platform. Enrichment of upregulated (***C***) and downregulated (***G***) molecular processes (C and G), elucidating the 10 main processes. Scatterplot of the analyses performed on the Enrichr platform, with the GO Molecular Process 2025 database. Each point represents a term in the library. Term frequency‐inverse document frequency (TF‐IDF) values ​​were computed for the gene set corresponding to each term, and uniform manifold approximation and projection (UMAP) was applied to the resulting values. Terms are plotted based on the first two dimensions of UMAP. Terms were automatically identified with the Leiden algorithm. Graphs of interaction of terms from the HMDB_Metabolites gene set analyzed in the Enrichr platform of upregulated (***D***) and downregulated (***H***) targets; *n* = 5 per group. Statistical analyses associated with the corrected *P* value and –log10 of the corrected *P* value (***A***, ***B***, ***E*** and ***F***), *P* value and TF‐IDF (***C*** and ***G***), and *P* value and odds ratio (***D*** and ***H***).

From the perspective of downregulated DEP enrichment, we observed the following biological pathways: Complement and coagulation cascades, Vesicle‐mediated transport, Biological oxidations, Post‐translational protein phosphorylation, Regulation of actin cytoskeleton, Metabolism, Innate immune system, Signalling by Rho GTPases, Focal adhesion, Muscle contraction, Hemostasis, and Metabolism of proteins (Fig. 2*E*); cellular components: Extracellular space, Endopeptidase inhibitor activity, Cytoplasm, Identical protein binding, Negative regulation of endopeptidase activity, Protease binding, Acute‐phase response, Extracellular region, Complement activation, classical pathway, Enzyme binding, Positive regulation of apoptotic cell clearance, and Myosin II complex (Fig. 2*F*); molecular parameters: Apolipoprotein receptor binding, Dipeptidase activity, Protease binding, Thioesterase binding, Phospholipase inhibitor activity, Calcium ion binding, Glutathione transferase activity, Myosin heavy chain binding, Myosin binding, and Cadherin binding (Fig. 2*G*); and metabolites: Hydrochloric acid standard solution, Sulfate, Glutathione, Bilirubin, C26H41NO34S4, UGA, Guanosine triphosphate, Retinoic acid, 9‐*cis*‐Retinal, and Estrone (Fig. 2*H*).

Subsequently, we identified the protein–protein interaction and annotated keywords (UniProt) enrichment of DEP upregulated (Fig. 3*A* and *B*) and DEP downregulated (Fig. 3*C* and *D*). We found 14 clusters of upregulated DEP, associated with Transit peptide, Mitochondrion, Acetylation, Oxidoreductase, Tricarboxylic acid cycle, Glycolysis, Methylation, Oxidation, Peroxidase, and ADP‐ribosylation (Fig. 3*A* and *B*); and 10 clusters of downregulated DEP, associated with Thioester bond, Secreted, Muscle protein, Acetylation, Complement pathway, Phosphoprotein, Intermediate filament, Myosin, Protease inhibitor, and Acute phase (Fig. 3*C* and *D*).

**Figure 3:**
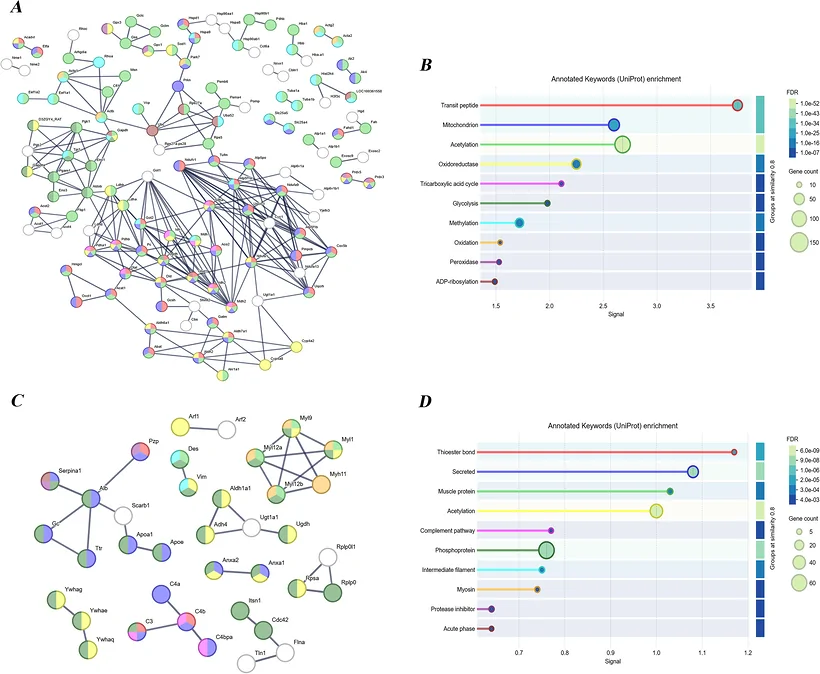
Protein–protein interaction network and annotated keywords (UniProt) enrichment of DEP upregulated (A and B) and downregulated (C and D) at PND540 based on the renal proteomic profile (n = 5 per group) Interactions of the identified proteins were mapped by searching the STRING database with a full STRING network and a 0.900 highest confidence. In the resulting protein association network, proteins are presented as nodes (A and C) that are connected by lines whose thickness represents the confidence level. The annotated keyword (Uniprot) enrichment (B and D) was analyzed considering FDR and gene count.

In addition, we confirmed the major interaction associated specifically with biochemical parameters, such as energetic metabolism. Using the protein–protein interaction of both upregulated and downregulated DEP, we observed the enrichment of Citrate cycle (TCA cycle), 2‐Oxocarboxylic acid metabolism, Pyruvate metabolism, Glycolysis/Gluconeogenesis, Carbon metabolism, Biosynthesis of amino acids, Parkinson disease, Prion disease, Pathways of neurodegeneration–multiple diseases, and Metabolic pathways (Fig. 4A), focusing on the metabolic pathways (Fig. 4*B*), specifically with TCA cycle, Pyruvate metabolism, and Glycolysis/Gluconeogenesis (Fig. 4*C–E*).

**Figure 4:**
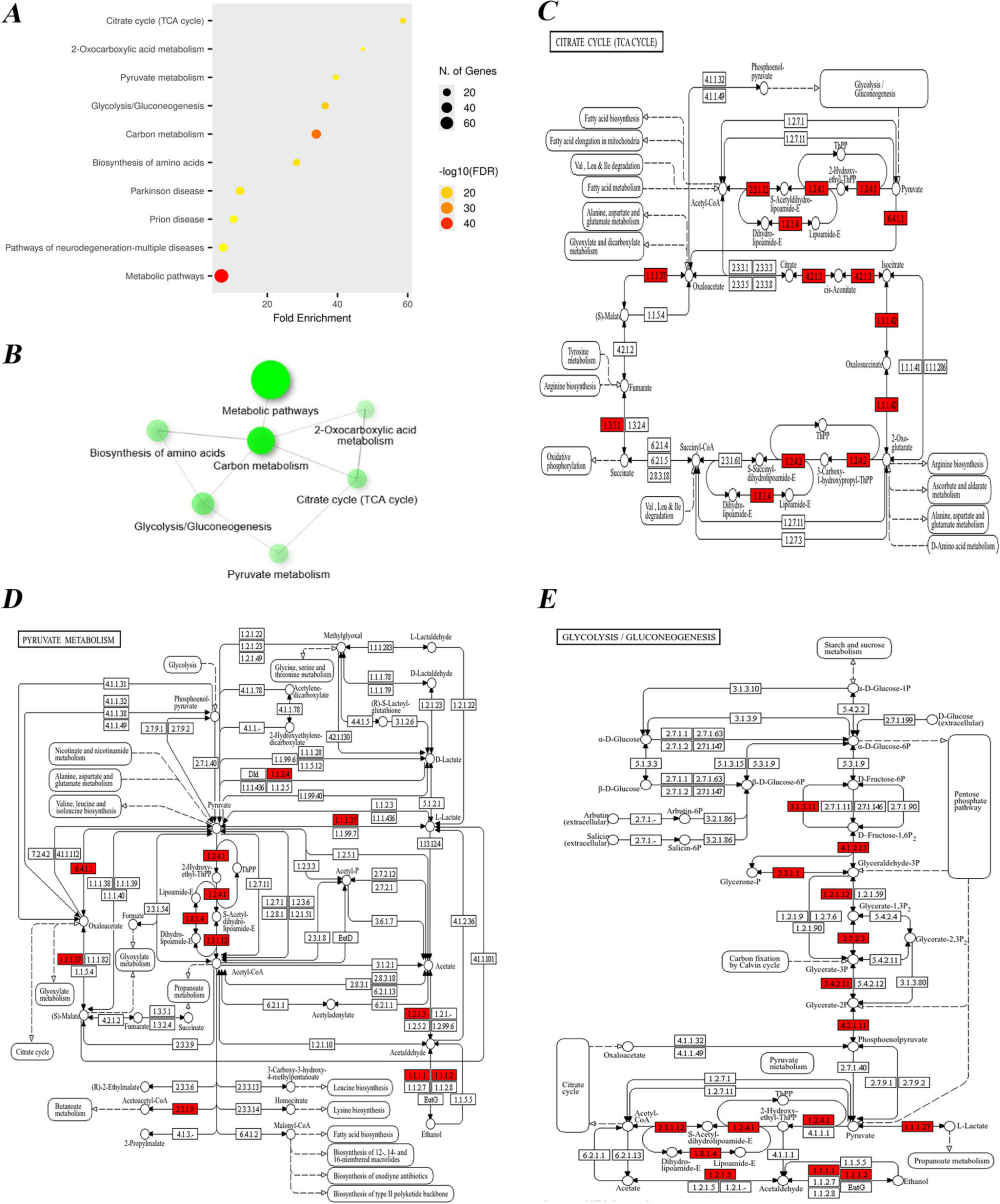
Integrated analysis of metabolic pathway enrichment and metabolic mapping (*n* = 5 per group) ***A***, showing the enrichment of metabolic pathways identified in the dataset. The *x*‐axis represents relative enrichment, the bubble size indicates the number of genes associated with each pathway, and the colour intensity corresponds to –log10 (FDR). ***B***, network representation of enriched pathways, illustrating the functional relationships between central metabolic processes. The node size reflects the significance of the enrichment, whereas the edges indicate the interconnections between pathways. ***C***–***E***, KEGG pathway maps highlighting the main metabolic routes affected in the dataset: Citrate cycle (TCA cycle) (***C***), Pyruvate metabolism (***D***) and Glycolysis/Gluconeogenesis (***E***). The red boxes indicate detected enzymes (DEP) associated with the analyzed gene/protein set, demonstrating the involvement of central carbon metabolism and energy‐related pathways.

Subsequently, we observed the main interaction between DEPs and miRNAs to identify possible epigenetic mechanisms associated with the effects of MPR on the kidneys during ageing (Fig. 5*A*). From these 57 specific protein targets, we found enrichment of chromosomal factors (Fig. 5*B*), exons (Fig. 5*C*), and biochemical and metabolic contexts: Lysine degradation, Pyruvate metabolism, Citrate cycle, Glycolysis and Gluconeogenesis, Valine leucine degradation, Urea cycle and metabolism of arginine proline glutamate aspartate and asparagine, Arginine proline metabolism, Glutamate glutamine metabolism, Methionine cysteine metabolism, and Glycine serine metabolism (Fig. 5*D*); Glyoxylate and dicarboxylate metabolism, Glycine serine and threonine metabolism, 2‐Oxocarboxylic acid metabolism, One carbon pool by folate, Carbon metabolism, Cysteine and methionine metabolism, Glycolysis/Gluconeogenesis, Biosynthesis of amino acids, Biosynthesis of cofactors, and Metabolic pathways (Fig. 5*E*).

**Figure 5:**
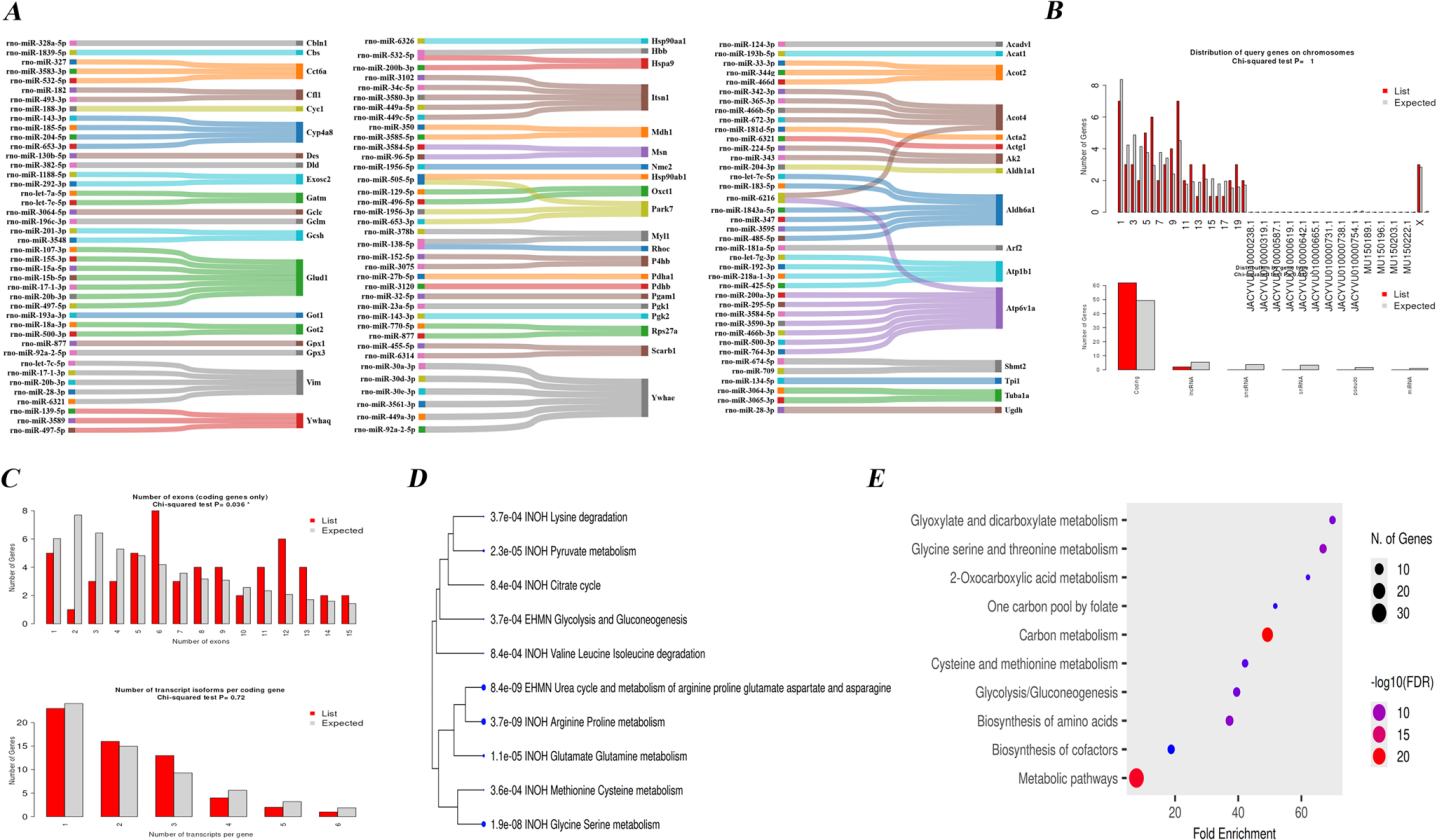
Integrated miRNA–epigenetic‐gene interaction and functional enrichment analysis of the potential epigenetic correlation of DEP (*n* = 5 per group) ***A***, Sankey diagrams illustrating the predicted interactions between DEP and validated or predicted miRNAs. The left and right panels represent the relationships between miRNAs and their corresponding gene targets, highlighting potential epigenetic regulation. ***B***, chromosomal distribution of the identified target DEP‐genes. The bar plot compares the observed number of genes (DEP) in the analyzed list (red) with the expected genomic distribution (grey), with statistical significance evaluated by the chi‐squared test. ***C***, genomic structure characteristics of the identified DEP‐genes, including the distribution of exon numbers per gene and the number of transcript isoforms per coding gene. Observed values (red) are compared with expected genomic frequencies (grey). ***D***, hierarchical clustering of enriched metabolic pathways based on functional similarity, highlighting major metabolic modules associated with amino acid metabolism, energy metabolism and central carbon metabolism. ***E***, bubble plot showing pathway enrichment analysis. The *x*‐axis represents fold enrichment, bubble size indicates the number of genes associated with each pathway, and colour intensity corresponds to –log10 (FDR). The analysis highlights pathways related to central carbon metabolism, amino acid biosynthesis, and glyoxylate and dicarboxylate metabolism.

In analyses of DEP enrichment with epigenetic potential and metabolic or renal diseases, we observed a correlation with metabolic syndrome, congenital hereditary anaemia, dysregulation of muscle and connective tissue, nephrosis, portal hypertension, organic acidosis, hypoglycemia, amino acid metabolism disorder, mitochondrial metabolism disease, and glycogen storage disease (Fig. 6*A* and *B*).

**Figure 6:**
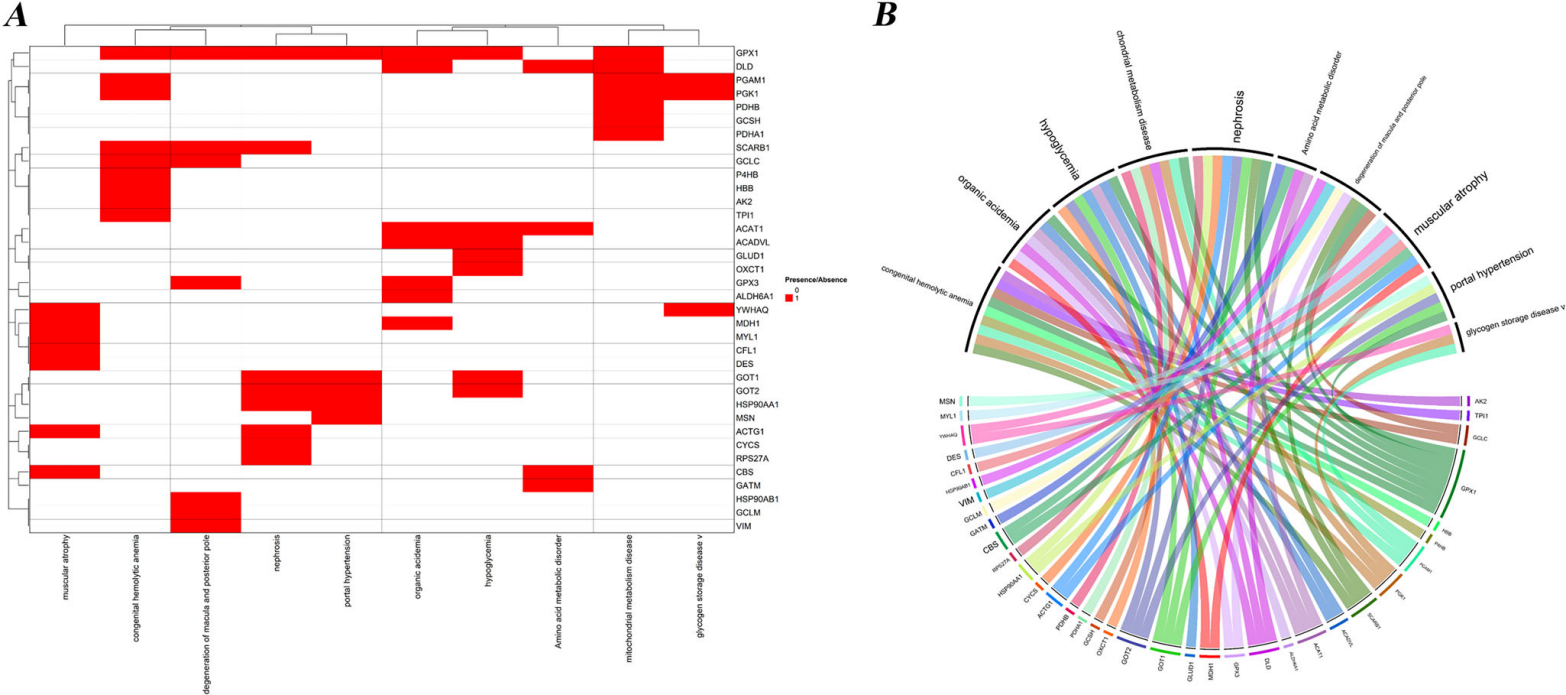
Integration of disease‐related signatures between identified epigenetic potential DEP (*n* = 5 per group) ***A***, heatmap showing the association between selected DEP and associated diseases with kidney or metabolic function. Red squares indicate the presence of known associations between each protein and the respective pathological condition, as curated from disease databases, including gene sets from DISEASES, GWAS, clusterProfiler, GSEABase and tidyverse. Hierarchical clustering was applied to proteins and diseases to highlight patterns of relationships. ***B***, circus plot illustrating the network of associations between identified proteins (below) and diseases (above). Colored ribbons represent known or predicted links extracted from the same integrated databases, revealing multidisease associations. Analyses were conducted in R, ComplexHeatmap and circlize. Statistical significance was assessed using the Benjamini–Hochberg method to correct for multiple testing, with a significance threshold of *P* < 0.05, based on the binary distance of the dendrograms.

After a more specific look at DEPs with epigenetic‐diseases‐metabolic enrichment, we observed interactions between these DEPs with the formation of five clusters: one associated with energy metabolism, one with fatty acid metabolism, one with stress and protein modifications, one with cytoskeleton organization and another with signal transduction (Fig. 7*A*). These targets reflect specific subcellular localization, specifically associated with mitochondria and cytoplasm (Fig. 7*B*). Visualizing the potential metabolites related to these DEPs, we observed enrichment and interactions with citric acid, oxaloacetate, NADP, NADH, glycine, l‐glutamate and ATP, among others (Fig. 7*C*). Finally, the enrichment of metabolic pathways continued to emphasize general processes already observed previously, such as cholesterol metabolism, the TCA cycle (Krebs cycle), hexose metabolism in the proximal tubules, retinol metabolism and inflammatory pathways (Fig. 7*D*). In general, the 16 DEPs emphasize the effects of MPR on energy metabolism, the Krebs cycle, amino acid and lipid metabolism, redox processes, and effects on signalling pathways and, primarily, mitochondrial function. There were 14 upregulated DEP and two downregulated DEP (YWHAQ and YWHAE).

**Figure 7:**
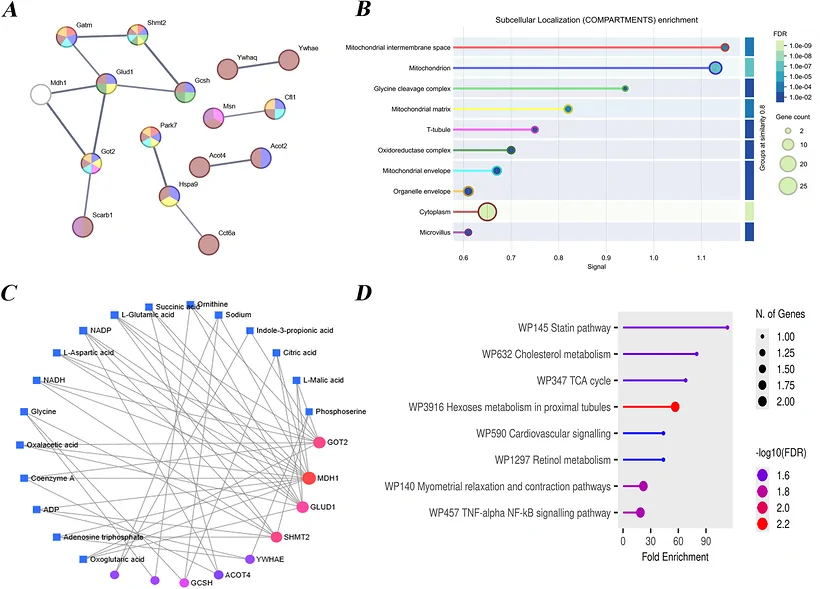
Protein–protein interaction of DEP epigenetic potential, subcellular localization (compartments) enrichment (A and B) metabolites enrichment (C) and associated pathways (D); n = 5 per group ***A***, interactions of the identified proteins were mapped by searching the STRING database with a full string network and a 0.900 highest confidence. The proteins are presented as nodes that are connected by lines whose thickness represents the confidence level. ***B***, subcellular localization (Compartments) enrichment was analyzed considering FDR and gene count. ***C***, enrichment of the specific metabolites associated with these DEPs, analyzed via the MetaboAnalyst6.0 platform. ***D***, hierarchical clustering of enriched metabolic pathways based on functional similarity, highlighting major metabolic modules.

Finally, we observed the kidney‐nephron‐specific expression of the main targets associated with epigenetic potential. Our results demonstrated that the targets GATM, SHMT2, GCSH, GLUD1, MDH1, GOT2, PARK7, HSPA9, MSN, CFL1, YWHAQ and YWHAE have high expression in the nephron epithelium; GCSH, GLUD1, MDH1, GOT2, PARK7, HSPA9, CFL1, YWHAQ and YWHAE in the ureteric epithelium; MDH1, SCARB1, PARK7, HSPA9, MSN, CFL1, YWHAQ and YWHAE in the vasculature; GATM, MDH1, PARK7, HSPA9, MSN, CFL1, YWHAQ and YWHAE in immune cells; and GCSH, GLUD1, MDH1, GOT2, PARK7, HSPA9, MSN, CFL1, YWHAQ and YWHAE in interstitial cells (Fig. 8*A–Q*). Intriguingly, however, we observed that CCT6B, ACOT4 and ACOT2 have very low expression in these regions (Fig. 8*K*, *L* and *M*). It is important to note that the platform used did not have data for CCT6B, and therefore we used the CCT6A isoform for this analysis.

**Figure 8:**
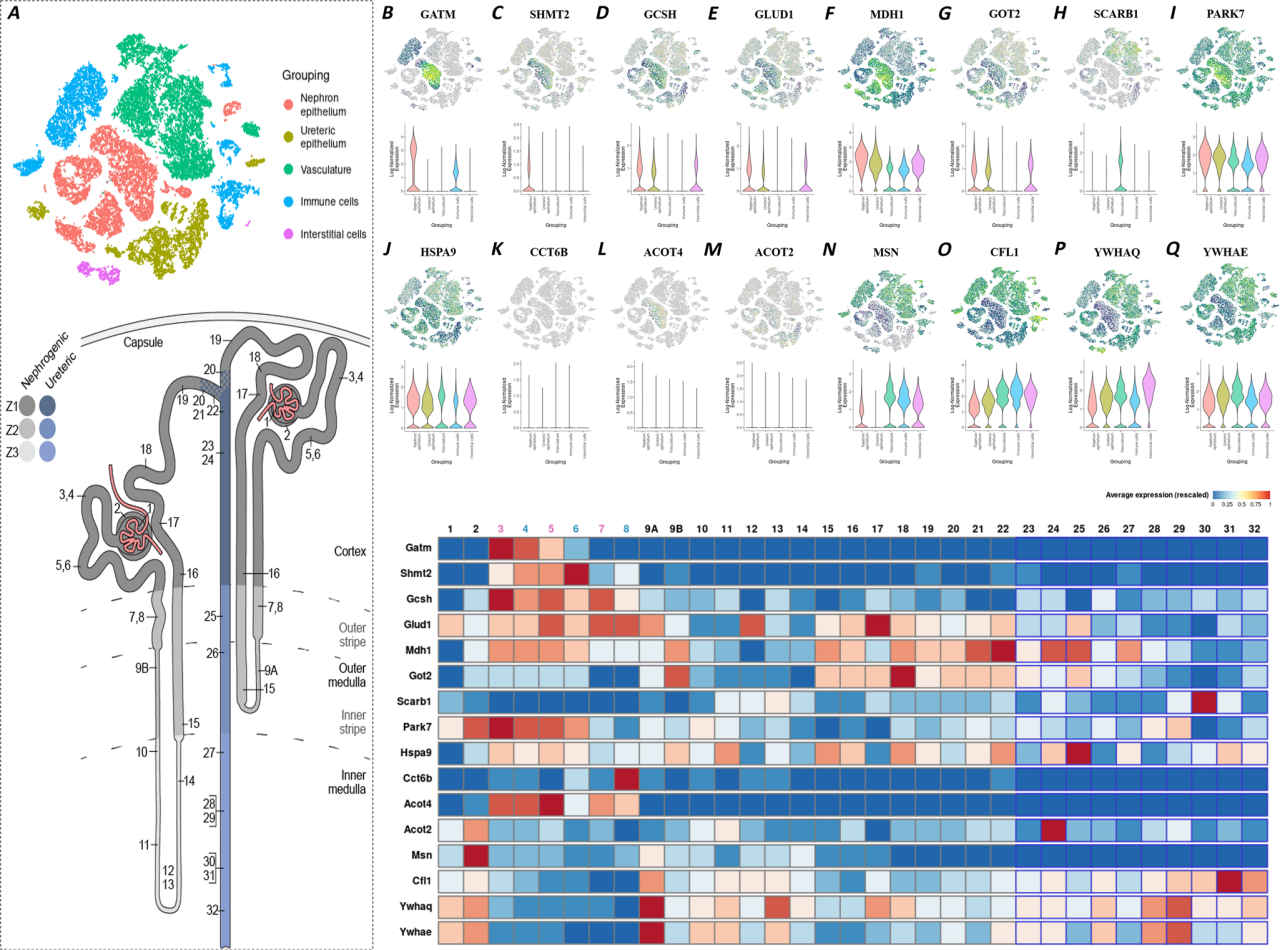
Single‐cell analysis (n = 5 per group) of GATM (B), SHMT2 (C), GCSH (D), GLUD1 (E), MDH1 (F), GOT2 (G), SCARB1 (H), PARK7 (I), HSPA9 (J), CCT6B (K), ACOT4 (L), ACOT2 (M), MSN (N), CFL1 (O), YWHAQ (P) and YWHAE (Q) targets in the kidneys and nephrons based on the selected DEPs dysregulated in GLLP animals The map elucidating the general regions of the urinary system, a specific view of the functional units (nephrons), the most enriched and expressed regions in the urinary system, the graph plotting the most enriched and expressed regions in the urinary system, and the degree of expression of the seven DEPs specifically in the nephron and collecting duct cells are shown (***A***). Analysis performed on the Kidney Cell Explorer platform. Statistical analyses based on normalization values ​​of the normalized log expression, with mean expression (scaled). 1: podocytes (visceral epithelium); 2: parietal epithelium; 3: proximal tubule segment 1 – female; 4: proximal tubule segment 1 – male; 5: proximal tubule segment 2 – female; 6: proximal tubule segment 2 – male; 7: proximal tubule segment 3 – female; 8: proximal tubule segment 3 – male; 9A: LOH thin descending limb of the inner stripe of the outer medulla of the cortical nephron; 9B: LOH thin descending limb of the inner strip of outer medulla of the juxtamedullary nephron; 10: superior LOH thin descending limb of the inner medulla of the juxtamedullary nephron; 11: inferior LOH thin descending limb of the inner medulla of the juxtamedullary nephron; 12: inferior LOH thin limb of the inner medulla of the juxtamedullary nephron; 13: inferior LOH thin limb of the inner medulla of the juxtamedullary nephron; 14: superior LOH thin ascending limb of the inner medulla of the juxtamedullary nephron; 15: distal straight tubule of inner strip of outer medulla (syn: thick ascending limb of LOH); 16: distal straight tubule of outer strip of outer medulla and cortex (syn: thick ascending limb of LOH); 17: mdensa; 18: distal convoluted tubule; 19: connecting tubule of nephron; 20: principal‐like cell of the connecting tubule of the nephron; 21: intercalated type non‐A non‐B cell of the connecting tubule of the nephron; 22: intercalated type A cell of the connecting tubule of the nephron and cortical collecting duct; 23: principal‐like cell of the cortical collecting duct; 24: intercalated type B cell of the cortical collecting duct; 25: intercalated type A cell of the outer medullary collecting duct; 26: principal cell of the outer medullary collecting duct; 27: intercalated type A cell of the inner medullary collecting duct; 28: principal cell of the inner medullary collecting duct type 1; 29: principal cell of the inner medullary collecting duct type 2; 30: principal‐like cell of the deep inner medullary collecting duct type 1; 31: deep inner medullary collecting duct type 2 cell; 32: deep medullary epithelium of the pelvis. Data from Kidney Cell Explorer (https://cello.shinyapps.io/kidneycellexplorer/; Ransick et al., 2019).

Specifically regarding the degree of expression in the nephron, GATM, SHMT2, GCSH, GLUD1, MDH1, PARK7, HSPA9, ACOT4, MSN, YWHAQ and YWHAE showed high or very high expression in the proximal convoluted tubules; GLU1, GOT2, HSPA9, CCT6B, ACOT4, CFL1, YWHAQ and YWHAE showed medium to high expression in the portions of the loop of Henle; GLUD1, MDH1, GOT2, HSPA9 and YWHAQ showed high expression in the distal convoluted tubule; and GLUD1, MDH1, GOT2, SCARB1, HSPA9, ACOT2, CFL1, YWHAQ and YWHAE showed medium to high expression in the collecting duct (Fig. 8). In our model, SCARB1, YWHAQ and YWHAE were downregulated in the kidneys of animals subjected to MPR, whereas CFL1, GATM, GCSH, GLUD1, GOT2, HSPA9, MDH1, MSN, PARK7, ACOT2, ACOT4, SHMT2 and CCT6A were upregulated.

## Discussion

The present study has examined how MPR affects the whole kidneys of ageing male rats, using both experimental and *in silico* multiomics approaches. We observed significant metabolic and histopathological changes, particularly in bioenergetic pathways and solute transport, which are linked to molecular, epigenetic and nephron‐segment–specific mechanisms. Incorporating proteomic data improves disease characterization. Several protein biomarkers, including CFL1, GATM, GLUD1, GOT2, HSPA9, MDH1, PARK7, SCARB1, ACOT2, SHMT2 and CCT6A, may serve as early indicators of renal changes, particularly mitochondrial and cytoplasmic dysregulation in nephron regions prone to tubular impairment. These biomarkers may be useful for disease monitoring and intervention.

From both metabolic and histopathological perspectives, our findings indicate that maternal protein restriction (MPR) induces persistent renal alterations, evidenced by elevated circulating creatinine and glycoprotein levels, along with renal congestion, tubular atrophy and areas of cellular vacuolization. In parallel, structural remodeling of the renal parenchyma was observed, characterized by reduced reticular fibre content (collagen III) and increased fibrotic deposition (collagen I). Interestingly, in patients with CKD and type 2 diabetes, an increase greater than 30% in the albumin/creatinine ratio in urine was associated with a higher risk of these clinical outcomes (Tangri et al., 2025).

The literature shows that tubular atrophy is also a characteristic of chronic kidney disease, a condition associated with a reduction in glomerular filtration rate, and the mechanisms involved in tubular atrophy include apoptosis of tubular epithelial cells, cellular senescence, peritubular capillary rarefaction and distal tubular ischaemia, oxidative stress, atubular glomeruli, epithelial‐mesenchymal transition, interstitial inflammation, lipotoxicity, and inactivation of the Na(+)/H(+)‐1 exchanger (Schelling, 2016). Experimental data further reinforce the relationship between tubular atrophy and renal fibrosis in models of severe ischaemia–reperfusion injury (Yamashita et al., 2020). Interestingly, epidemiologically, it is well documented that an adverse intrauterine environment is associated with an increased risk of hypertension and kidney disease in adulthood (Luyckx & Brenner, 2015). In a model of renal tubulointerstitial fibrosis, adult rats exposed to maternal food restriction during gestation showed increased tubular necrosis in the obstructed kidney, in addition to fibrosis (Awazu et al., 2019). In addition, folate deficiency increased inflammation and exacerbated renal fibrosis in mice fed a high‐fat, high‐fructose diet (Chan & Lin, 2023). Collectively, these alterations suggest that early‐life protein restriction promotes long‐lasting disruption of renal architecture and extracellular matrix organization, establishing a tissue microenvironment prone to progressive renal dysfunction and increased susceptibility to kidney disease later in life.

In the biological, cellular, molecular and metabolite enrichment, upregulated DEPs indicated an exacerbation of small molecule transport, energy metabolism, carbohydrate and amino acid metabolism, and stress response, comprising conditions directly associated with mitochondrial function, oxidation–reduction aspects and electron transport. On the other hand, downregulated DEPs indicated mechanisms of cellular regulation, the immune system, biological oxidation, and vesicular transport, comprising conditions directly associated with the cytoskeleton, extracellular responses, molecular bonds and glutathione. Rats exposed to MPR showed impaired renal development and ciliogenesis during fetal life, from mechanisms that promoted apoptosis in renal tubular epithelial cells (Wang et al., 2023).

Also, during fetal life, other authors observe that MPR (9%) causes adverse effects on sodium regulation mechanisms and response to angiotensin in male rats (Alwasel et al., 2010; Torreggiani et al., 2020), evidencing a possible short‐term effect on cellular transport processes, small molecule transport and renal cellular regulatory capacity. Later during development into adulthood, MPR increases the glomerular filtration rate after salt overload (Hoppe et al., 2007). Lamana et al. (2021) observed that, in adulthood, rodents exposed to MPR showed intense sodium reabsorption in the proximal segments of the nephron. Associated with our results, it is plausible to consider that MPR impacts renal processes and functional capacity throughout life, which is reflected in exacerbated mechanisms of transport during ageing. This can be associated with increased sodium reabsorption and higher blood pressure.

From a general proteomic data perspective, MPR leads to premature renal ageing, which can be understood from the beginning of life by the decrease in the number of nephrons, leading to a state of hyperfunction of filtration and reabsorption capacity, characterized by increased anaerobic metabolism (LDHA and LDHB), higher glycolytic activity and gluconeogenesis (FBP1, ALDOB, TPI1, GAPDH, PGJ1, PGAM, ENO1 and ENO3), higher pyruvate to acetyl‐CoA conversion (DLAT, PDHA1 and PDHB) and increased activity of the Krebs cycle (ACO2, IDH1, IDH2, OGDH and MDH2) and the electron transport chain (NDUFA9, NDUFA13, NDUFV1, SDHA, VQCRC1, CYC1, UQCRH, COX5B, ATP5F1B and ATP5F1A).

On the other hand, this metabolic overload also reveals imbalances in the recycling and reorganization of different macromolecules, through the increased conversion of oxaloacetate to pyruvate (FAHD1) and pyruvate to oxaloacetate (PC), in addition to the activity of the malate‐aspartate shuttle (MDH1 and GOT1). Still within the metabolic aspect, the renal proteomic profile indicates increased metabolism of long‐chain fatty acids and β‐oxidation (ACOT1, ACOT2, ACOT4, ETFA and ACADVL), as well as the metabolism of amino acids (GATM, ALDH6A1, ALDH7A1, ALDH2, CBS, SHMT2, GCSH and HGD) and components of the urea cycle (GOT2). This scenario indicates a very evident metabolic disorder and a condition of overload of renal functions, which may be associated with adverse conditions such as response to hypertension (acid‐base imbalance) and renal hyperfunction in the face of renal injury. The increase in ATP1B1, ATP1A1, SLC25A5, SLC25A4, ATP5Po, ATP6V1A and ATP6V1B1 is an important indicator of this acid–base imbalance, which may be associated with tubular alkalosis and tubulointerstitial fibrosis. These cellular alterations also reflect antioxidant impacts, specifically through the increase in GCLC, GCLM, GPX1, GPX3, SOD1, GSS and PARK7. Furthermore, we observed downregulation of transporters such as apolipoproteins (APOA1 and APOE), D vitamin (GC), ALB, HDL receptor (SCARB1) and thyroid hormone transporters (TTR). Furthermore, some downregulations in metabolic mechanisms show renal impacts (ADH4, ALDH1A1, UGT1A1 and UGDH); for example, associated with ketone bodies (OXCT1). Taken together, the results indicate that the identified miRNAs may regulate an integrated set of genes primarily involved in energy and amino acid metabolism, suggesting a regulatory network with potential impact on central metabolic processes and cellular homeostasis.

Specifically, the effects of MPR appear to upregulate aspects of acetylation, oxidation–reduction, energy metabolism and mitochondrial function in the kidneys of ageing male rats, whereas the capacity for cellular regulation, cytoskeleton‐dependent transport and secretion processes, and even muscle contraction appear to be impaired. This scenario encompasses classic stress characteristics, which may include hormone‐dependent dysregulation, effects of renal structural damage or even metabolic diseases that impair this organ. The most interesting finding is that the literature shows male renal alterations in response to MPR, similar to those seen in our analyses, but mainly in fetal and adult life, highlighting the potential long‐term effects. For example, Gomes et al. (2023) observed that MPR reduced nephron number in male fetus, a condition related to changes in the HIF‐1α signalling pathway, NOS, Ep300 and HSP90 expression. Male rats exposed to MPR (10%) during pregnancy and lactation demonstrated reduction in the number of nephrons and renal blood flow, as well as vasoactive, antioxidant and anti‐inflammatory molecules, although they showed resilience to acute kidney injury during adult life (García‐Ortuño et al., 2019). Guo et al. (2020) showed that MPR modulates the overall proteomic profile of fetal kidneys, enriching biological pathways associated with ion channels/transporters, reducing the expression of aquaporins 2/4, causing an imbalance in solute transporter isoforms (SLCs), as well as increasing the expression of pathways associated with apoptosis and increasing ATP6V1C2, with some molecules also found in our model, with patterns of expression differences. An experimental study evaluated the effects of gestational and lactational MPR or neonatal overnutrition on young male animals, and observed increased lipid peroxidation, as well as reduced glutathione, total thiol content, and renal superoxide dismutase and catalase activities (Pedroza et al., 2019). These data are quite interesting because they reveal disturbances in the antioxidant system and metabolic aspects in the kidneys of animals exposed to MPR, but still in the early stages of life.

Furthermore, structurally, our analyses present interesting results. MPR affects renal cellular dynamism in numerous ways, impacting the organization of the cytoskeleton (CFL1 and MSN), general cell actins (ACTB and ACTG1) and general cell tubulins (TUBA1A and TUBA1B), as well as contractile proteins (MYl9, MYL1, MYL12a and MYL12b) and associated proteins (VIM and DES); interestingly, smooth muscle actins, which may be associated with an increased response of the juxtaglomerular apparatus and renin response (increased ACTG2, ACTA2 and decreased MYH11). The increase in ACTA2 is quite interesting because it is a smooth muscle protein that indicates activation of renal mesangial cells, but which, on the other hand, can identify phenotypic changes in the glomeruli, indicating a scenario of injury or stress, a plausible mechanism considering the observed histopathological lesions.

Furthermore, from the perspective of cellular homeostasis, numerous mechanisms are altered in relation to catalytic activities and vesicular transport (increased RHOA, RHOC and ARHGDIA; decreased ARF1, ARF2 and ITSN1), as well as the activation of protein repair and degradation machinery (VCP, UBC, RPS5, RPS27A, PRKN, PSMB6, PSMA4, POMP, UBA52, CCT6A, P4HB and HSP isoforms) and RNA catabolism (EXOSC9 and EXOSC2). On the other hand, decreased mechanisms associated with phospholipid‐binding membrane proteins and the anti‐inflammatory response (ANXA1 and ANXA2), as well as the complement system (C3, C4B, C4A and C4BPA), were observed. Furthermore, our results elucidated a decrease in factors associated with cell–cell and extracellular matrix interaction (TLN1) and mediate signal transduction by binding to phosphoserine‐containing proteins (YWHAG, YWHAE and YWHAQ). Given this, the structural impact on the kidneys is also quite evident, influencing the responsiveness of renal cells, secretory activity, and even basic filtration and reabsorption mechanisms, indicated by intracellular stress, hyperactivation of protein repair machinery and inactivation of the complement system, which, associated with the metabolic scenario, certainly suggests a scenario of renal injury.

From a DOHaD perspective, Alvarez et al. (2026) demonstrate that MPR induces early‐life renal impairment in male rats, accompanied by widespread proteomic reprogramming. This includes a decrease in energy metabolism, ion transport, cytoskeletal organization, membrane integrity and mitochondrial function, alongside the upregulation of pathways related to innate immunity, glutathione metabolism, vesicular trafficking and cytoskeletal dynamics. Notably, we elucidated key differentially expressed proteins, such as GPX1, CYCS, ATP1A2/ATP1B1, TUBB/TUBA isoforms, ANPEP and multiple metabolic enzymes, that emerge as potential biomarkers of renal metabolic and epigenetic programming. Strikingly, several targets dysregulated in early life persist into ageing, either maintaining their altered expression or exhibiting inversion patterns. Among these, MDH1, GOT2 and GLUD1 are particularly noteworthy, because they are consistently identified as biomarkers across the lifespan. This temporal overlap reveals a dynamic reprogramming process in which specific biological pathways, especially those related to energy metabolism, undergo marked reversal during ageing.

It is essential to clarify that the role of miRNA expression is always inversely related to the expression of DEPs. As examples of potential miRNAs in renal programming and their derivations in response to the MPR and ageing, we can cite miR107‐3p, which regulates GLUD1 expression and has already been associated as a target to inhibit proliferation, fibrosis and progression of diabetic nephropathy (Zhang et al., 2024). miR15a‐5p, which also regulates GLUD1, has been identified through *in silico* analyses as related to the pathogenesis of sepsis‐induced acute kidney injury (Xu et al., 2019). On the other hand, miR‐18a‐3p, associated with GOT2 expression, has also been reported to be involved in the pyroptosis processes of renal tubular epithelial cells in lipopolysaccharide‐induced acute kidney injury models (Deng et al., 2021). Another interesting target is miR‐130b‐5p, which interacts with DES – downregulated, and a study evaluating urinary exosomes from people with type 2 diabetes mellitus and diabetic kidney disease showed that the expression of miR‐30b‐5p was significantly lower (Zang et al., 2019). MYL1 and RHOC appear associated with miR‐138‐5p, and their upregulated miR‐138‐5p inhibits proliferation and invasion in certain kidney tumour types (Liu & Qu, 2021). On the other hand, miR‐143‐3p (associated with CYP4A8 expression) was a promising target identified in mesenchymal stem cells for attenuating acute kidney injury (Xu et al., 2025), whereas another study associated this same miRNA with attenuating diabetic kidney disease (Song et al., 2026). Interestingly, the fact that CYP4A8 is overexpressed in our model, indicating fatty acid omega‐hydroxylase, considered a biomarker of autosomal recessive polycystic kidney disease and hypertension, indicates low expression of miR‐143‐3p, which may demonstrate a decrease in protective non‐coding factors that can lead to a risk of kidney disease. Another interesting target is miR‐20b‐3p, which regulates both GLUD1 and VIM, and protects against kidney stones by suppressing autophagy and inflammatory responses (Shi et al., 2019). Another promising target associated with the regulation of GLUD1 and YWHAQ was miR‐497‐5p, which has previously been associated with chemoresistance of renal cell carcinoma (Jin et al., 2020). In our analyses, we observed that GPX1 and RPS27A were correlated with miR‐877. A very relevant piece of data was reported by Xie et al. (2017), who found that patients with type 2 diabetes with macroalbuminuria had an increase in the miRNAs miR‐362‐3p, miR‐877‐3p and miR‐150‐5p derived from exosomes, considering them as biomarkers for the early diagnosis of diabetic kidney disease. Other *in vitro* work has elucidated that miR‐92a‐2‐5p and its regulated pathways promote renal fibrosis (Xie et al., 2024), whereas Tsai et al. (2023) showed, both *in vitro* and *in vivo*, that this same miRNA derived from proximal tubule exosomes contributes to mesangial cell damage in diabetic nephropathy. In our data, this miRNA is related to the epigenetic potential and regulation of GPX3 and YWHAE.

Using a non‐human primate model exposed to prenatal gestational and early childhood caloric restriction, a previous study demonstrated that offspring presented elevated fasting insulin levels at 6 months of age, in addition to increased serum osteocalcin levels and decreased bone mineral density (Kirigiti et al., 2020). Although this study does not fully emphasize renal function, it is quite intuitive to integrate the observation that the alterations in our model may also indicate a renal inability to maintain calcium, either as a result of transport defects or hormone‐dependent factors, which certainly generates a risk for the development of neurological and bone disorders. Another study conducted with rodents elucidated that adult offspring exposed to prenatal MPR and maternal caloric restriction presented increased microalbuminuria, decreased glomerular filtration rate and hypertension (Almeida & Mandarim‐de‐Lacerda, 2005). Studies have shown that rats exposed to gestational MPR develop arterial hypertension and long‐term hyperfiltration in adulthood, evidenced by proteinuria (Sene et al., 2018). Low birth weight is one of the consequences of MPR, and the literature explains that this condition can generate a tendency and risk for CKD and hypertension (Kanda et al., 2020). Experimental data support that MPR increases the risk of developing hypertension, a condition associated with disturbances in nephrogenesis (Harrison & Langley‐Evans, 2009) and renal response (e.g. renin–angiotensin–aldosterone system) (Woods et al., 2001). These data are also associated with the increased risk of diseases observed in our analyses, such as hypoglycemia, portal hypertension, mitochondrial metabolic disease and amino acid metabolism disorders.

In the specific results of the major interactions, we again highlighted the main impacts on mitochondrial or cytoplasmic aspects associated with oxidation‐reduction (NADH, NADP and ADP). We were again able to demonstrate the enrichment of the TCA cycle and hexose metabolism in the proximal tubules, which indicates a direct impact on the main reabsorption site. Looking specifically at some of these targets, the literature elucidates that GATM is associated with creatine biosynthesis, and the literature shows that its dysregulation may be related to various conditions of renal dysfunction (Medeiros et al., 2025; Reichold et al., 2018). Another quite interesting study, using integrated analysis of plasma proteome and transcriptome data, identified GATM and 31 other potential therapeutic targets associated with CKD (Si et al., 2024). Our analyses also showed an increase in SHMT2, a key enzyme in one‐carbon metabolism that converts serine into a one‐carbon unit linked to tetrahydrofolate and glycine, and that has also previously been associated with renal cell ferroptosis and interstitial fibrosis (Lyu et al., 2025). We also observed increased expression of GCSH, a crucial component of the mitochondrial glycine cleavage system, and a classic study elucidated that non‐ketotic hyperglycinemia, an inborn error of metabolism (deficiency in the glycine cleavage system), may be associated with alterations in GCSH (Kure et al., 2001). The evident increase expression of these three proteins in the proximal convoluted tubule may indicate an overload in the physiological reabsorption processes in response to injury, or even an attempt to compensate for glomerular damage and the excess of proteins/amino acids present in the filtrate.

Other important overexpressed targets were GOT2, MDH1 and GLUD1, targets related to amino acid metabolism, the urea cycle and the malate‐aspartate shuttle. GOT2, for example, has already been associated with inflammatory aspects and clear cell renal carcinoma (Ferreira et al., 2022). GLUD1 has been related to hypoxia and impaired renal response (oxidative injury and apoptosis (He et al., 2020). MDH1 is little studied in the real sense, although its mitochondrial isoform MDH2 has been related to cellular senescence and reoxygenation (Eleftheriadis et al., 2022). In general, these three molecules indicate an overload or compensatory mechanism of the nephron to metabolize amino acids and perhaps eliminate urea, at the same time as it leads to an attempt to move more cytoplasmic NADH to the mitochondria, a factor that can be associated with the need for greater ATP in the face of renal overload or even a less functional kidney.

ACOT4, for example, a key regulator of fatty acid metabolism, has already been reported as protective against kidney injury (Wang et al., 2025), but ACOT2 has also been correlated as a genetic determinant of blood metabolites in CKD in a Chronic Kidney Disease Cohort Study (Rhee et al., 2022). CFL1 is necessary for maintaining mature podocyte architecture (Garg et al., 2010), which is interesting because it is overexpressed in our model, perhaps as an adaptive response. Experimental data show that PARK7 protects against chronic kidney injury and renal fibrosis (Pfister et al., 2024; Yin et al., 2021), another interesting finding that may indicate DEP overexpression in response to systemic and tissue injury. Other targets, such as SCARB1, associated with reverse cholesterol transport, and HSPA9, a chaperone protein, have already been reported in different types of renal tumours (Ruzza et al., 2025; Song et al., 2023). On the other hand, YWHAQ and YWHAE, related to signal transduction, are relatively new proteins in the context of renal dysregulation because the literature does not trace their direct effects on kidney function. Specifically, Specifically, YWHAE (14‐3‐3 epsilon) and YWHAQ (14‐3‐3 tau or theta) are members of the 14‐3‐3 protein family involved in stabilizing actin and vimentin filaments to maintain podocyte processes (Yasuda et al., 2023). Additionally, the 14‐3‐3 complex interacts with synaptopodin in the podocyte actin cytoskeleton, which is essential for glomerular barrier integrity (Faul et al., 2008). These two proteins are downregulated, whereas, in parallel, CFL1, comprising a protein that binds to F‐actin and promotes its depolymerization, is overexpressed. Thus, it can be hypothesized that these alterations lead to the rearrangement of the podocyte actin cytoskeleton, resulting in subsequent proteinuria. The cytoplasmic tail of podoplanin, a podocyte membrane protein, interacts with ezrin, radixin and MSN proteins to promote epithelial‐mesenchymal transition (Martín‐Villar et al., 2006). Furthermore, the upregulation of MSN in the parietal and visceral epithelia of Bowman's capsule suggests glomerular epithelial‐to‐mesenchymal transition (EMT). A study using a very similar model has previously demonstrated glomerular EMT in adult rats (Sene et al., 2013).

Fioretto et al. (2025) demonstrated, for example, that animals exposed to MPR have increased corticosterone, which reflects a catabolic scenario that may correlate with changes in amino acid metabolism, whereas there is a decrease in aldosterone levels, which certainly alters the acid–base balance and also sodium regulation, an effect that may be a response to hypertension, as already observed in these animals during adult life (Mesquita et al., 2010). Our findings demonstrate that the long‐term effects of MPR persist into ageing, manifesting as metabolic overload, oxidative imbalance and disrupted transport processes, all of which are accompanied by histopathological alterations and may increase susceptibility to renal disease. Proteomic analyses indicate upregulation of pathways related to acetylation, redox homeostasis, energy metabolism and mitochondrial function, whereas processes involved in cellular regulation, cytoskeleton‐dependent transport, secretion and muscle contraction are downregulated. These alterations suggest a chronic stress state associated with structural renal damage, metabolic dysregulation and endocrine‐dependent mechanisms. Nephron‐segment mapping further indicates that MPR predominantly affects the glomerulus, proximal convoluted tubule, distal convoluted tubule and collecting duct, supporting impairments in glomerular filtration, tubular metabolic activity and hormone‐dependent renal function. Finally, MPR appears to promote both aerobic and anaerobic metabolic overload, accompanied by activation of antioxidant, protein repair and cellular stress pathways, highlighting persistent metabolic and redox adaptations in the ageing kidney.

This study has several limitations. First, we did not perform direct functional assessments of renal physiology, such as glomerular filtration rate, tubular transport, renal clearance or urinary biomarkers, limiting functional confirmation of the observed alterations. Second, because this was a proteomics‐driven study, mechanistic validation relied primarily on *in silico* analyses, and further molecular and functional validation is warranted. Third, our analyses were restricted to male offspring. This choice was based on previous evidence demonstrating that maternal protein restriction preferentially reduces nephron number and predisposes males to renal dysfunction and hypertension. Nevertheless, the absence of female data remains an important limitation. Although females are also expected to be affected, sex‐specific developmental and epigenetic mechanisms, together with the protective effects of female sex hormones, may differentially modulate renal programming. Future studies should therefore investigate these sex‐dependent responses. Finally, although the rat is a well‐established model for developmental programming, species‐specific differences should be considered when translating these findings to humans. In addition, only maternal protein restriction was evaluated; future studies should examine other nutritional insults, including caloric restriction, high‐fat diets and postnatal environmental adversities. Despite these limitations, our findings provide a strong foundation for understanding the developmental origins of renal dysfunction induced by maternal malnutrition and for guiding future mechanistic, translational and clinical studies.

From a translational perspective, our severe MPR (6% protein) model represents an isolated reduction in protein intake that may resemble conditions of severe food insecurity or famine; however, it does not encompass the full complexity of human malnutrition. In clinical settings, maternal malnutrition is highly heterogeneous and commonly involves moderate protein deficiency combined with energy and micronutrient deficiencies. Therefore, future clinical and epidemiological studies should investigate the real human nutritional insults within the DOHaD framework to better define their effects on embryo‐fetal programming and the long‐term risk of renal disease.

## Conclusions

In conclusion, this study demonstrates that MPR induces whole‐kidney programming consistent with the DOHaD paradigm, thereby increasing the lifelong risk of renal dysfunction. Our findings show that progressive and persistent metabolic, histopathological, proteomic, cellular, molecular and epigenetic changes affect tubular energy metabolism and nephron‐specific protein expression, leading to functional disorders. Identifying biomarkers linked to MPR‐induced programming may help prevent diagnostic errors and reduce the risk of progression for renal and metabolic diseases. Given the complexity of mammalian kidneys, studying isolated glomeruli and distinct nephron segments may provide a more precise approach to identifying abnormalities caused by early gestational malnutrition. These results improve understanding of the long‐term impact of early‐life malnutrition on renal health and clarify the biological pathways linking early nutritional insults to age‐related renal dysfunction.

## Additional information

## Competing interests

The authors declare that they have no competing interests.

## Author contributions

M.N.F., M.P.P. and L.A.J. were responsible for conceptualization, data curation, formal analysis, validation, funding acquisition and writing the original draft. M.N.F., M.P.P., L.A.J., I.T.R., L.A.B., F.A.M., L.S.L., M.R.F., R.M., L.M.F.P., P.V.S., L.C.G. and G.M.C. were responsible for investigations. M.N.F., M.P.P., L.A.J., I.T.R., L.A.B., F.A.M. and L.S.L. were responsible for the methodology. All authors were responsoble for reviewing and editing.

## Funding

This study was financed in part by the Coordenação de Aperfeiçoamento de Pessoal de Nível Superior – Brasil (CAPES 88 887.112689/2025‐00 to MNF); The São Paulo Research Foundation (FAPESP) (2024/22 188‐6 to MNF; 2023/0 6148‐1 to MPP; 2025/0 1977‐5 to WRS; 2022/0 3990‐0 and 2025/21 512‐7 to LAJ); Conselho Nacional de Desenvolvimento Científico e Tecnológico (310 663/2018‐0); CNPq/INCT DOHaD (Proc #409 165/2024‐7).

## Generative AI statement

All the text of the manuscript was initially written without Generative AI. Generative AI (ChatGPT) was partially used to correct some English formulations or to improve the formulation of a few sentences.

## Supporting information




Peer Review History

Supporting Information

## Data Availability

All data are provided within the final article.
