# Supplementary material for: Maternal protein restriction programs the ageing kidney: Proteomic signatures of early‐life origin renal and metabolic dysfunction
Source: J Physiol. 2026 Aug 31;604(20):8636–61. doi: 10.1113/JP291758 (PMC13674207; doi:10.1113/JP291758)
Supplement: Supplementary file 1 [file TJP-604-8636-s002.pdf]

The Journal of Physiology

Maternal Protein Restriction Programs the Aging Kidney: Proteomic Signatures of Early-Life Origin Renal and Metabolic Dysfunction

Marina Pereira Pires, Matheus Naia Fioretto, Marcel Rodrigues Ferreira, Isabelle Tenori Ribeiro, Luisa Annibal Barata, Flávia Alessandra Maciel, Lucas Sobrinho Lemos, Renato Mattos, Luiz Marcos Frediani Portela, Patrick Vieira Souza, Luisa Correia Gomes, Gustavo Monezzi Cordeiro, Wellerson Rodrigo Scarano, Patricia Aline Boer, Jose Antonio Rocha Gontijo, and Luis Antonio Justulin
DOI: 10.1113/JP291758

Corresponding author(s): Luis Justulin (l.justulin@unesp.br)

The following individual(s) involved in review of this submission have agreed to reveal their identity: John F Bertram (Referee #1)

Review Timeline:

Submission Date:	14-May-2026
Editorial Decision:	02-Jul-2026
Revision Received:	13-Jul-2026
Accepted:	31-Jul-2026

Senior Editor: Kim Barrett

Reviewing Editor: Janna Morrison

Transaction Report:

(Note: With the exception of the correction of typographical or spelling errors that could be a source of ambiguity, letters and reports are not edited. Depending on transfer agreements, referee reports obtained elsewhere may or may not be included in this compilation. Referee reports are anonymous unless the Referee chooses to sign their reports.)

Re: JP-RP-2026-291758 "**Maternal Protein Restriction Programs the Aging Kidney: Proteomic Signatures of Early-Life Origin Renal and Metabolic Dysfunction**" by Marina Pereira Pires, Matheus Naia Fioretto, Marcel Rodrigues Ferreira, Isabelle Tenori Ribeiro, Luisa Annibal Barata, Flávia Alessandra Maciel, Lucas Sobrinho Lemos, Renato Mattos, Luiz Marcos Frediani Portela, Patrick Vieira de Souza, Luisa Correia Gomes, Gustavo Monezzi Cordeiro, Wellerson Rodrigo Scarano, Patricia Aline Boer, Jose Antonio Rocha Gontijo, and Luis Antonio Justulin

Dear Dr Justulin,

Thank you for submitting your manuscript to The Journal of Physiology. It has been assessed by a Reviewing Editor and by 2 expert referees and we are pleased to tell you that it is potentially acceptable for publication following satisfactory major revision.

Please advise your co-authors of this decision as soon as possible.

The referee reports are copied at the end of this email.

Please address all the points raised and incorporate all requested revisions or explain in your Response to Referees why a change has not been made. We hope you will find the comments helpful and that you will be able to return your revised manuscript within 2 months. If your article is NOT for a Special Issue, you may have 9 months to revise. If you require an extension, please contact journal staff: jp@physoc.org. Please note that this letter does not constitute a guarantee for acceptance of your revised manuscript.

Your revised manuscript should be submitted online using the link in your Author Tasks: Link Not Available. This link is accessible via your account as Corresponding Author; it is not available to your co-authors. If this presents a problem, please contact journal staff (jp@physoc.org). Image files from the previous version are retained on the system. Please ensure you replace or remove any files that are being revised.

If you do not wish to submit a revised version of your manuscript, you must inform our journal staff (jp@physoc.org) or reply to this email to request withdrawal. Please note that a manuscript must be formally withdrawn from the peer review process at one journal before it may be submitted to another journal.

TRANSPARENT PEER REVIEW POLICY: To improve the transparency of its peer review process, The Journal of Physiology publishes online as supporting information the peer review history of all articles accepted for publication. Readers will have access to decision letters, including Editors' comments and referee reports, for each version of the manuscript, as well as any author responses to peer review comments. Referees can decide whether or not they wish to be named on the peer review history document.

ABSTRACT FIGURES: Authors are expected to use The Journal's premium BioRender account to create/redraw their Abstract Figures. Information on how to access this account is here: https://physoc.onlinelibrary.wiley.com/journal/14697793/biorender-access.

This will enable Authors to create and download high-resolution figures. If authors have used the free BioRender service, they can use the instructions provided in the link above to download a high-resolution version suitable for publication.

The link provided should only be used for the purposes of this submission. Authors will be charged for figures created on this account if they are not related to this manuscript submission.

LANGUAGE EDITING AND SUPPORT FOR PUBLICATION: If you would like help with English language editing, or other article preparation support, Wiley Editing Services offers expert help, including English Language Editing, as well as translation, manuscript formatting, and figure formatting at www.wileyauthors.com/eeo/preparation. You can also find resources for Preparing Your Article for general guidance about writing and preparing your manuscript at www.wileyauthors.com/eeo/prepresources.

RIGOUR AND REPRODUCIBILITY: We recommend authors consult The Journal's Rigour and Reproducibility requirements to ensure that their manuscript is suitable for publication: https://physoc.onlinelibrary.wiley.com/pb-assets/hub-assets/physoc/documents/TJP-Rigour-and-Reproducibility-Requirements.pdf

REVISION CHECKLIST:

Check that your Methods section conforms to journal policy: https://jp.msubmit.net/cgi-bin/main.plex?form_type=display_requirements#methods.

Check that data presented conforms to the statistics policy: https://jp.msubmit.net/cgi-bin/main.plex?form_type=display_requirements#statistics.

Upload a full Response to Referees file. To create your 'Response to Referees': copy all the reports, including any

comments from the Senior and Reviewing Editors, into a Microsoft Word, or similar, file and respond to each point, using font or background colour to distinguish comments and responses and upload as the required file type.

Please upload two versions of your manuscript text: one with all relevant changes highlighted and one clean version with no changes tracked. The manuscript file should include all tables and figure legends, but each figure/graph should be uploaded as separate, high-resolution files.

You may also upload:

- 'Potential Cover Art' for consideration as the issue's cover image
- Appropriate Supporting Information (Video, audio or data set: see https://jp.msubmit.net/cgi-bin/main.plex?form_type=display_requirements#supp).

We look forward to receiving your revised submission.

If you have any queries, please reply to this email and we will be pleased to advise.

Yours sincerely,

Kim Barrett
Senior Editor
The Journal of Physiology

REQUIRED ITEMS

- Author photo and profile. First or joint first authors are asked to provide a short biography (no more than 100 words for one author or 150 words in total for joint first authors) and a portrait photograph. These should be uploaded and clearly labelled together in a Word document with the revised version of the manuscript. See Information for Authors for further details.

- The Journal of Physiology funds authors of provisionally accepted papers to use the premium BioRender site to create high resolution schematic figures. Follow this link and enter your details and the manuscript number to create and download figures. Upload these as the figure files for your revised submission. If you choose not to take up this offer, we require figures to be of similar quality and resolution. If you are opting out of this service to authors, state this in the Comments section on the Detailed Information page of the submission form. The link provided should only be used for the purposes of this submission. Authors will be charged for figures created on this premium BioRender account if they are not related to this manuscript submission.

- Please upload separate high-quality figure files via the submission form.

- Please ensure that any tables are editable and in Word format, and wherever possible, embedded in the article file itself.

- Your paper contains Supporting Information of a type that we no longer publish, including supplementary tables and figures. Any information essential to an understanding of the paper must be included as part of the main manuscript and figures. If inclusion of this material would interrupt the 'flow' of the paper, it can be included at the bottom as part of an Appendix, instead. Any figures and tables should be numbered as Fig. A1, Fig. A2, Table A1, etc. - and each should be cited in the text. The only Supporting Information that we publish are video and audio, 3D structures, program codes and large data files. Your revised paper will be returned to you if it does not adhere to our Supporting Information Guidelines.

- Papers must comply with the Statistics Policy: https://jp.msubmit.net/cgi-bin/main.plex?form_type=display_requirements#statistics.

In summary:

- If n {less than or equal to} 30, all data points must be plotted in the figure in a way that reveals their range and distribution. A bar graph with data points overlaid, a box and whisker plot or a violin plot (preferably with data points included) are acceptable formats.

- If n > 30, then the entire raw dataset must be made available either as supporting information, or hosted on a not-for-profit repository, e.g. FigShare, with access details provided in the manuscript.

- 'n' clearly defined (e.g. x cells from y slices in z animals) in the Methods. Authors should be mindful of pseudoreplication.

- All relevant 'n' values must be clearly stated in the main text, figures and tables.

- The most appropriate summary statistic (e.g. mean or median and standard deviation) must be used. Standard Error of the Mean (SEM) alone is not permitted.

- Exact p values must be stated. Authors must not use 'greater than' or 'less than'. Exact p values must be stated to three significant figures even when 'no statistical significance' is claimed.

- A Data Availability Statement is required for all papers reporting original data. This must be in the Additional Information section of the manuscript itself. It must have the paragraph heading 'Data Availability Statement'. All data supporting the results in the paper must be either: in the paper itself; uploaded as Supporting Information for Online Publication; or archived in an appropriate public repository. The statement needs to describe the availability or the absence of shared data. Authors must include in their statement: a link to the repository they have used, or a statement that it is available as Supporting Information; reference the data in the appropriate sections(s) of their manuscript; and cite the data they have shared in the References section. Whenever possible, the scripts and other artefacts used to generate the analyses presented in the paper should also be publicly archived. If sharing data compromises ethical standards or legal requirements then authors are not expected to share it, but must note this in their statement. For more information, see our Statistics Policy.

- Please include an Abstract Figure legend. An appropriate figure legend, which should not exceed 150 words in length, should be included in the main manuscript file. All Abstract Figures should be created using BioRender. Authors should use The Journal's premium BioRender account to export high-resolution images. Details on how to use and access the premium account are included as part of this email.

EDITOR COMMENTS

Please also see 'Required Items' above.

Reviewing Editor: Comments to the Author (Required):
Thank you for submitting this interesting manuscript.

Why were only males studied?

The sample size of dams is noted in the methods. Please indicate the number of pups used for each analysis.

Was more than one pup per litter used in an analysis? eg histology. If yes, was the analysis nested for dam?

Senior Editor:

Comments for Authors to ensure the paper complies with the Statistics Policy (Required):
Please check our statistical policy for guidance on required data presentation, including the provision of individual data points and precise p values.

Comments to the Author:
The authors are asked to review our policy for supplemental information. This must be restricted to large datasets; other information (e.g., the first supplemental item) should be incorporated in the body of the manuscript

----

REFEREE COMMENTS

Referee #1:

The developmental programming of adult diseases is now an accepted paradigm. Much previous work has focused on the developmental programming of kidney-related diseases including hypertension and chronic kidney disease. Numerous reports of reduced nephron endowment and renal pathophysiology have been published. However, the cellular and molecular mechanisms whereby events in early life (during gestation and/or early after birth) impact kidney structure and function in later life have not been extensively studied. This study provides perhaps the most thorough analyses to date on the molecular, genetic and epigenetic mechanisms that result in impaired kidney structure following sub-optimal kidney development.

The study investigated the effects of maternal protein restriction on kidney health in offspring at postnatal day 540. Six control diet male rats and 6 protein-restricted male rats were studied. The study reports findings on the renal proteome, combined with integrated histological , molecular and in silico assays. The number and originality of the findings are impressive and set a new standard in investigating the mechanisms and consequences involved in developmental programming of the kidney. It will be interesting to see in future studies whether the kidney changes reported here are also seen in other models of developmental programming of the kidney.

There are several limitations to the study. The major limitation is that female rats were not studied. However, given the enormous amount of information and original data already in this manuscript, I think this limitation can be justified. Only one model of developmental programming (gestational and lactational maternal protein restriction) was studied, but again this is not a major flaw. Moreover, no detailed analysis of renal physiology (GFR, blood pressure etc) were conducted.

Specific Comments.

1. Some references in the text include the surname of more than one author.

2. The fat and carbohydrate contents of the diets should be provided, not just the protein concentration.

3. Define kidney "congestion".

4. Many terms in Methods were followed by definitions or explanations. These are not necessary. Examples include: "Mast cells (a type of immune cell)"; "rotary microtome (an instrument for slicing tissue)"; "acetonitrile (an organic solvent)"; "upregulated (increased)"; Downregulated (decreased)". There a several more.

5. I could. not find the "Naia Fioretto et al 2024 reference in the bibliography.

6. Table 1. Units are required for many of the parameters.

7. Section 2.6 Statistical Analysis says data are "expressed as mean +/- SD". However, data in Table 1 are expressed as mean +/- SE.

Referee #2:

Pires and Fioretto et al. investigate how maternal protein restriction (MPR) impacts the adult kidney. It provides valuable insight into how MPR, which is highly prevalent among women of childbearing age in developing countries, impacts renal function and expands the knowledge base regarding DoHad. They show that MPR can increase the risk of renal dysfunction in aging male kidneys, which sheds light on how early life exposure can program the increased risk for hypertension. The authors utilised a wide variety of advanced molecular techniques to understand the metabolic, histopathological, proteomic,

cellular, and epigenetic changes that occur in response to MPR. Overall, the manuscript is well written, and the authors declare that they used ChatGPT to improve English correctness.

I have listed some points below that require attention.

1. Terms should be stated in full upon first mention. For instance, MPR in the key points summary (line 34), PND540 in the abstract (line 48), gene names in methods and AST and ALT (lines 361, table 1). In other instances, some terms are abbreviated inconsistently (such as chronic kidney disease).

2. The key points summary could be expanded upon.

3. It is unclear at times when the authors are referring to the fetal kidney or adult kidney, such as in the abstract (Lines 44-46) and introduction (Lines 93-96). Please clarify.

4. For transparency, the abstract should state what species of rat and the severity of MPR utilised in this study (global, moderate or severe, state percentage reduction).

5. Whilst I commend the authors for their meticulous reporting of their molecular methods. The methods section is dense. This is sometimes attributed to the authors defining terms that aren't critical to the reproducibility of the experiment; for example, they define terms such as rotary microtome (line 153), H&E (line 156), and mast cell (line 163). Please consider cutting unnecessary words to streamline the information and improve readability.

6. The authors state that the animals received gestational and lactational MPR; however, offspring are raised until postnatal day 540. Did the pups return to the control diet post-weaning? This should be stated.

7. Line 143: Where was the blood collected from?

8. The authors are transparent that this study exclusively used male offspring for molecular analysis. What was the fate of female offspring? It is unclear if they were also euthanised. This should be made clear.

9. The statistical analysis section states that data is expressed as mean {plus minus} standard deviation. However, the Table 1 legend states that the data are expressed as mean {plus minus} standard error.

10. Table 1 should present all P values for transparency. Consider adding a 4th column to display P values.

11. Figure 1 demonstrates the effort made by the authors to understand the histology of these kidneys. However, the graphs are small and hard to see relative to the representative histology images.

12. The authors have made great attempts to interpret their data in the discussion. Some concepts feel repetitive and could be merged to streamline the message and condense this section (Line 738-41, 840-846, 911-923)

13. The authors acknowledge that the use of only males is a limitation, as the responses reported may be sex-specific. In addition, the authors should provide a brief justification for the exclusive use of males. Furthermore, what phenotypes might the authors have expected in female adult kidneys? The literature shows that hypertension in offspring born to poor in utero

environments can be male-predominant.

14. In addition to my above comments, authors should be cautious not to generalise findings as it is not appropriate to directly compare the adult data to both male and female adult kidneys. Where possible, when referencing outcomes from other studies, state whether the previous findings were sex-specific or in both sexes (Lines 670-680, 699, 719-736, 769).

15. The authors could justify the use of a 6% protein restriction, which is considered severe. Does the severity of MPR reflect that experienced globally in women? Discussion on how this might compare to a moderate MPR or a diet restricted in more substrates (global) may be useful.

END OF COMMENTS

Pires and Fioretto et al. investigate how maternal protein restriction (MPR) impacts the adult kidney. It provides valuable insight into how MPR, which is highly prevalent among women of childbearing age in developing countries, impacts renal function and expands the knowledge base regarding DoHad. They show that MPR can increase the risk of renal dysfunction in aging male kidneys, which sheds light on how early life exposure can program the increased risk for hypertension. The authors utilised a wide variety of advanced molecular techniques to understand the metabolic, histopathological, proteomic, cellular, and epigenetic changes that occur in response to MPR. Overall, the manuscript is well written, and the authors declare that they used ChatGPT to improve English correctness.

I have listed some points below that require attention.

1. Terms should be stated in full upon first mention. For instance, MPR in the key points summary (line 34), PND540 in the abstract (line 48), gene names in methods and AST and ALT (lines 361, table 1). In other instances, some terms are abbreviated inconsistently (such as chronic kidney disease).

2. The key points summary could be expanded upon.

3. It is unclear at times when the authors are referring to the fetal kidney or adult kidney, such as in the abstract (Lines 44-46) and introduction (Lines 93-96). Please clarify.

4. For transparency, the abstract should state what species of rat and the severity of MPR utilised in this study (global, moderate or severe, state percentage reduction).

5. Whilst I commend the authors for their meticulous reporting of their molecular methods. The methods section is dense. This is sometimes attributed to the authors defining terms that aren't critical to the reproducibility of the experiment; for example, they define terms such as rotary microtome (line 153), H&E (line 156), and mast cell (line 163). Please consider cutting unnecessary words to streamline the information and improve readability.

6. The authors state that the animals received gestational and lactational MPR; however, offspring are raised until postnatal day 540. Did the pups return to the control diet post-weaning? This should be stated.

7. Line 143: Where was the blood collected from?

8. The authors are transparent that this study exclusively used male offspring for molecular analysis. What was the fate of female offspring? It is unclear if they

were also euthanised. This should be made clear.

9. The statistical analysis section states that data is expressed as mean ± standard deviation. However, the Table 1 legend states that the data are expressed as mean ± standard error.

10. Table 1 should present all P values for transparency. Consider adding a 4th column to display P values.

11. Figure 1 demonstrates the effort made by the authors to understand the histology of these kidneys. However, the graphs are small and hard to see relative to the representative histology images.

12. The authors have made great attempts to interpret their data in the discussion. Some concepts feel repetitive and could be merged to streamline the message and condense this section (Line 738-41, 840-846, 911-923)

13. The authors acknowledge that the use of only males is a limitation, as the responses reported may be sex-specific. In addition, the authors should provide a brief justification for the exclusive use of males. Furthermore, what phenotypes might the authors have expected in female adult kidneys? The literature shows that hypertension in offspring born to poor *in utero* environments can be male-predominant.

14. In addition to my above comments, authors should be cautious not to generalise findings as it is not appropriate to directly compare the adult data to both male and female adult kidneys. Where possible, when referencing outcomes from other studies, state whether the previous findings were sex-specific or in both sexes (Lines 670-680, 699, 719-736, 769).

15. The authors could justify the use of a 6% protein restriction, which is considered severe. Does the severity of MPR reflect that experienced globally in women? Discussion on how this might compare to a moderate MPR or a diet restricted in more substrates (global) may be useful.

Instituto de Biociências
Unesp Botucatu

July 13, 2026

To **Kim E. Barrett, PhD**

Editor-in-Chief of The Journal of Physiology

Dear Editor,

We are resubmitting our manuscript "*Maternal Protein Restriction Programs the Aging Kidney: Proteomic Signatures of Early-Life Origin Renal and Metabolic Dysfunction*" for possible publication, by consideration in the Editorial Board of The Journal of Physiology.

We thank the reviewers for the insightful comments, and several significant changes were made. All mandatory issues raised by the reviewers have been consistently addressed. We used yellow highlights to indicate new sentences and changes in the revised manuscript.

Thank you for your attention to this matter.

Sincerely yours,

Luis Antonio Justulin

Department of Structural and Functional Biology, Institute of Biosciences, São Paulo State University, Botucatu, São Paulo, Brazil, Zip Code: 510; P.O Box: 18618-689, Rubião Júnior, s/n, Botucatu, SP – Brazil, Phone: +55 (14) 3880-0481.

Email: l.justulin@unesp.br

Response to editor and Reviewers

EDITOR COMMENTS

Answer: We sincerely appreciate the opportunity provided by the Editors to revise our manuscript and thank the reviewers for their insightful comments, constructive criticisms, and valuable suggestions. We carefully considered all comments, thoroughly revised and restructured the manuscript accordingly, and addressed each point in detail in the responses below, with all corresponding modifications highlighted in the revised manuscript. We hope that these revisions have substantially strengthened the quality and clarity of our work and that the manuscript is now suitable for publication in The Journal of Physiology. We remain available to address any further questions or suggestions.

Reviewing Editor: Comments to the Author (Required):

Thank you for submitting this interesting manuscript.

Why were only males studied?

Answer: We agree that the lack of female analyses represents an important limitation, and this will certainly be addressed in our future studies. Initially, our research focused primarily on reproductive and prostatic outcomes following maternal protein restriction (MPR) (PMID: 37972683; PMID: 33049715; PMID: 37907720; PMID: 39639045; PMID: 36499183). More recently, we expanded our investigations to include systemic and metabolic mechanisms in male offspring, encompassing multiple organs and signaling pathways (PMID: 38880278; PMID: 39986598; PMID: 40032160; PMID: 42025250; PMID: 42091765; PMID: 41106765; PMID: 39218056). To specifically address sex-dependent developmental programming, we have now begun replicating these experimental models in female offspring, focusing on the liver, cardiovascular system, and hypothalamic-pituitary-thyroid-adrenal axis during early life (PMID: 42402324; PMID: 41407027; PMID: 40300334). We therefore fully acknowledge this limitation and anticipate that our ongoing studies will provide important insights into the sex-specific effects of MPR across multiple biological systems.

unesp
UNIVERSIDADE ESTADUAL PAULISTA
"JÚLIO DE MESQUITA FILHO"
Câmpus de Botucatu
(14) 3880 - 0481
l.justulin@unesp.br
R. Prof. Doutor Antonio Celso Wagner Zanin, 250
CEP: 18618-689 - Botucatu/SP, Brazil

The sample size of dams is noted in the methods. Please indicate the number of pups used for each analysis.

Answer: Thank you. Done.

Was more than one pup per litter used in an analysis? eg histology. If yes, was the analysis nested for dam?

Answer: Thank you very much for the excellent question. In fact, only one pup per litter from each dam was used for the analyses. Furthermore, at birth, litters were standardized to 8 pups per dam (1:1 male:female) to control for maternal behavioral bias. Litters with fewer than 8 pups or that did not meet the sex ratio were excluded. The remaining pups from the same dam were allocated to analyses for other projects or discarded.

Senior Editor:

Comments for Authors to ensure the paper complies with the Statistics Policy (Required):

Please check our statistical policy for guidance on required data presentation, including the provision of individual data points and precise p values.

Answer: Thank you for the reminder. We have adjusted Figure 1 to align with the journal's policies. Additionally, we have added p-values to Table 2.

Comments to the Author:

The authors are asked to review our policy for supplemental information. This must be restricted to large datasets; other information (e.g., the first supplemental item) should be incorporated in the body of the manuscript

Answer: Thank you very much. We have incorporated the first supplementary item (Table) into the body of the revised manuscript.

----

(14) 3880 - 0481
l.justulin@unesp.br

R. Prof. Doutor Antonio Celso Wagner Zanin, 250
CEP: 18618-689 - Botucatu/SP, Brazil

REFEREE COMMENTS

Referee #1:

The developmental programming of adult diseases is now an accepted paradigm. Much previous work has focused on the developmental programming of kidney-related diseases including hypertension and chronic kidney disease. Numerous reports of reduced nephron endowment and renal pathophysiology have been published. However, the cellular and molecular mechanisms whereby events in early life (during gestation and/or early after birth) impact kidney structure and function in later life have not been extensively studied. This study provides perhaps the most thorough analyses to date on the molecular, genetic and epigenetic mechanisms that result in impaired kidney structure following sub-optimal kidney development.

Answer: We sincerely appreciate the reviewer's thoughtful, constructive, and insightful comments. We fully agree that the primary objective of this study was to provide a comprehensive characterization of the long-term effects of maternal protein restriction during gestation and lactation on the renal health of male rat offspring, integrating molecular, epigenetic, biochemical, proteomic, cellular, and histopathological approaches. The reviewer's valuable suggestions have helped us improve the quality, clarity, and scientific rigor of the manuscript. Accordingly, we have carefully addressed all comments through a thorough revision and restructuring of the manuscript, and we provide detailed point-by-point responses below, with all modifications clearly highlighted. We hope that the revised version is now suitable for publication in *The Journal of Physiology*, and we remain grateful for the opportunity to improve our work. We would be pleased to address any additional questions or concerns if required.

The study investigated the effects of maternal protein restriction on kidney health in offspring at postnatal day 540. Six control diet male rats and 6 protein-restricted male rats were studied. The study reports findings on the renal proteome, combined with integrated histological , molecular and in silico assays. The number and originality of the findings are impressive and set a new standard in investigating the mechanisms and consequences involved in developmental programming of the kidney. It will be interesting to see in future studies whether the kidney changes reported here are also seen in other models of developmental programming of the kidney.

Answer: Once again, we appreciate the reviewer's comments. Indeed, the study provides a solid, comprehensive foundation regarding the biological mechanisms—across various scales—by

(14) 3880 - 0481
l.justulin@unesp.br
R. Prof. Doutor Antonio Celso Wagner Zanin, 250
CEP: 18618-689 - Botucatu/SP, Brazil

which maternal protein restriction and embryonic, fetal, and neonatal programming affect the kidneys in the long term. Future studies aimed at understanding how other models of malnutrition (e.g., caloric or micronutrient deficiency) and different adverse postnatal environments (e.g., exposure to high-fat diets) impact long-term renal health will certainly offer important complementary insights into the effects of various exposures on kidney health. We hope this study serves as a basis for such future analyses.

There are several limitations to the study. The major limitation is that female rats were not studied. However, given the enormous amount of information and original data already in this manuscript, I think this limitation can be justified. Only one model of developmental programming (gestational and lactational maternal protein restriction) was studied, but again this is not a major flaw. Moreover, no detailed analysis of renal physiology (GFR, blood pressure etc) were conducted.

Answer: We appreciate the reviewer's excellent points. Indeed, the study's main limitation is the absence of assessments regarding females and sex-specific differences. This limitation will certainly be addressed in the group's future studies. Previously, we focused on analyses of reproductive and prostatic aspects (PMID: 37972683; DOI: 10.1016/j.mce).2023.112102; PMID: 33049715 PMCID: PMC7655221 DOI: 10.18632/aging.104093; PMID: 37907720 PMCID: PMC10618455 DOI: 10.1038/s41598-023-46068-1; PMID: 39639045 PMCID: PMC11621812 DOI: 10.1038/s41598-024-77901-w; PMID: 36499183 PMCID: PMC9739077 DOI: 10.3390/ijms232314855) and, more recently, we expanded this scope to include metabolic and systemic mechanisms, investigating the effects on male offspring (PMID: 38880278 DOI: 10.1016/j.mce.2024.112316; PMID: 39986598 DOI: 10.1016/j.yexcr.2025.114464; PMID: 40032160 DOI: 10.1016/j.cellsig.2025.111707; PMID: 42025250 DOI: 10.1016/j.clnu.2026.106656; PMID: 42091765 PMCID: PMC13149610 DOI: 10.1007/s13105-026-01189-9; PMID: 41106765 DOI: 10.1016/j.yexcr.2025.114795; PMID: 39218056 DOI: 10.1016/j.mce.2024.112348). To address these sex-specific differences, we have recently begun replicating the experiments, examining differences regarding liver health, the pituitary-thyroid-adrenal axis, and the heart, albeit still in early life (PMID: 42402324 DOI: 10.1016/j.yexcr.2026.115122; PMID: 41407027 DOI: 10.1016/j.mce.2025.112715; PMID:

UNIVERSIDADE ESTADUAL PAULISTA
"JÚLIO DE MESQUITA FILHO"
Câmpus de Botucatu
(14) 3880 - 0481
l.justulin@unesp.br
R. Prof. Doutor Antonio Celso Wagner Zanin, 250
CEP: 18618-689 - Botucatu/SP, Brazil

40300334 DOI: 10.1016/j.bbrc.2025.151873). Certainly, the limitation of the lack of analysis of females will be addressed in future studies by the group.

Furthermore, we agree that the lack of functional analyses concerning blood pressure, glomerular filtration rate, reabsorption, and secretion constitute a limitation, and we highlight all these points at the end of our discussion as study limitations.

"This study has several limitations. First, we did not perform direct functional assessments of renal physiology, such as glomerular filtration rate, tubular transport, renal clearance, or urinary biomarkers, limiting functional confirmation of the observed alterations. Second, as this was a proteomics-driven study, mechanistic validation relied primarily on in silico analyses, and further molecular and functional validation is warranted. Third, our analyses were restricted to male offspring. This choice was based on previous evidence demonstrating that maternal protein restriction preferentially reduces nephron number and predisposes males to renal dysfunction and hypertension. Nevertheless, the absence of female data remains an important limitation. Although females are also expected to be affected, sex-specific developmental and epigenetic mechanisms, together with the protective effects of female sex hormones, may differentially modulate renal programming. Future studies should therefore investigate these sex-dependent responses. Finally, although the rat is a well-established model for developmental programming, species-specific differences should be considered when translating these findings to humans. In addition, only maternal protein restriction was evaluated; future studies should examine other nutritional insults, including caloric restriction, high-fat diets, and postnatal environmental adversities. Despite these limitations, our findings provide a strong foundation for understanding the developmental origins of renal dysfunction induced by maternal malnutrition and for guiding future mechanistic, translational, and clinical studies."

Specific Comments

1. Some references in the text include the surname of more than one author.

Answer: Thank you, we have corrected them.

unesp UNIVERSIDADE ESTADUAL PAULISTA
"JÚLIO DE MESQUITA FILHO"
Câmpus de Botucatu

(14) 3880 - 0481
l.justulin@unesp.br

R. Prof. Doutor Antonio Celso Wagner Zanin, 250
CEP: 18618-689 - Botucatu/SP, Brazil

2. The fat and carbohydrate contents of the diets should be provided, not just the protein concentration.

Answer: Thank you very much for the suggestion. We have added this information to the body of the manuscript (Materials and Methods section), specifying that full details of the diets are provided in the supplementary material.

"...The normoprotein diet contained 627.5 g/kg of carbohydrates and 70 g/kg of lipids, whereas the low-protein diet contained 760 g/kg of carbohydrates and 70 g/kg of lipids. Both diets were isocaloric (3.96 kcal/g), with the lower protein content compensated by increased carbohydrate levels. The nutritional information is provided in Table 1."

3. Define kidney "congestion".

Answer: Thank you very much for the excellent point. Renal congestion is characterized by the dilation of vessels associated with the glomerulus and/or peritubular veins and capillaries, which may indicate functional alterations. We have added this information to the manuscript.

4. Many terms in Methods were followed by definitions or explanations. These are not necessary. Examples include: "Mast cells (a type of immune cell)"; "rotary microtome (an instrument for slicing tissue)"; "acetonitrile (an organic solvent)"; "upregulated (increased)"; Downregulated (decreased)". There a several more.

Answer: Thank you very much for the excellent point. We have removed those redundant passages from the methods section of the revised manuscript.

5. I could. not find the "Naia Fioretto et al 2024 reference in the bibliography.

Answer: Thank you. We have added the reference to the revised manuscript.

"Naia Fioretto, M., Maciel, F. A., Barata, L. A., Ribeiro, I. T., Basso, C. B. P., Ferreira, M. R., Dos Santos, S. A. A., Mattos, R., Baptista, H. S., Portela, L. M. F., Padilha, P. M., Felisbino, S. L., Scarano, W. R., Zambrano, E., & Justulin, L. A. (2024). Impact of maternal protein restriction on the proteomic landscape of male rat lungs across the lifespan. Molecular and cellular endocrinology, 592, 112348. https://doi.org/10.1016/j.mce.2024.112348"

(14) 3880 - 0481
l.justulin@unesp.br
R. Prof. Doutor Antonio Celso Wagner Zanin, 250
CEP: 18618-689 - Botucatu/SP, Brazil

6. Table 1. Units are required for many of the parameters.

Answer: Thank you. We have added this information to the revised Table 2 in the manuscript, as follows:

***"Table 2.** Biometric and metabolic parameters in the offspring at PND540.*

Parameters	***CTR***	***GLLP***	
Body mass (g)	*537.9±126.5*	*513.3±61.3*	*p=0.5380*
Kidney mass (g)	*4.121±0.6*	*3.726±0.6*	*p=0.1205*
AST (U/L)	*102.0±30.1*	*105.0±61.7*	*p=0.9037*
ALT (U/L)	*6.7±1.5*	*10.0±3.6*	*p=0.0620*
Creatinine (mg/dL)	*0.59±0.09*	*0.76±0.13**	*p=0.0337*
Alkaline phosphatase (U/L)	*281.6±77.69*	*391.0±101.0*	*p=0.0649*
Urea (mg/dL)	*22.53±3.672*	*23.62±1.796*	*p=0.2403*

**Statistically significant difference between control (CTR) and gestational and lactational protein restriction (GLLP). Data are presented as mean ± SEM (n=6/group). Statistical analysis by the "t" test, with significance when p-value < 0.05."*

7. Section 2.6 Statistical Analysis says data are "expressed as mean +/- SD". However, data in Table 1 are expressed as mean +/- SE.

Answer: Thank you for the comment. We have added the correct information to section 2.6, as follows:

"...Data were expressed as mean± standard deviation (SD) or Standard Error of the Mean (SEM)..."

Referee #2:

Pires and Fioretto et al. investigate how maternal protein restriction (MPR) impacts the adult kidney. It provides valuable insight into how MPR, which is highly prevalent among women of childbearing age in developing countries, impacts renal function and expands the knowledge base regarding DoHad. They show that MPR can increase the risk of renal dysfunction in aging male kidneys, which sheds light on how early life exposure can program the increased risk for hypertension. The authors utilised a wide variety of advanced molecular techniques to understand the metabolic, histopathological, proteomic, cellular, and epigenetic changes that occur in

unesp UNIVERSIDADE ESTADUAL PAULISTA
"JÚLIO DE MESQUITA FILHO"
Câmpus de Botucatu
(14) 3880 - 0481
l.justulin@unesp.br
R. Prof. Doutor Antonio Celso Wagner Zanin, 250
CEP: 18618-689 - Botucatu/SP, Brazil

response to MPR. Overall, the manuscript is well written, and the authors declare that they used ChatGPT to improve English correctness.

Answer: We sincerely appreciate the reviewer's insightful comments, constructive criticisms, and valuable suggestions. This study provides a comprehensive investigation of the long-term effects of maternal protein restriction (MPR) within the DOHaD framework, integrating renal histopathological, epigenetic, molecular, biochemical, and cellular analyses through a proteomics-based approach. We have carefully addressed all of the reviewer's comments by thoroughly revising and restructuring the manuscript, and all modifications are detailed in the point-by-point responses below. We believe these revisions have substantially strengthened the manuscript and hope it is now suitable for publication in The Journal of Physiology. We remain available to address any further questions or suggestions.

I have listed some points below that require attention.

1. Terms should be stated in full upon first mention. For instance, MPR in the key points summary (line 34), PND540 in the abstract (line 48), gene names in methods and AST and ALT (lines 361, table 1). In other instances, some terms are abbreviated inconsistently (such as chronic kidney disease).

Answer: Thank you. We have adjusted these terms and acronyms in the revised manuscript.

2. The key points summary could be expanded upon.

Answer: Thank you for the excellent suggestion. We have expanded on our key points in the revised manuscript, as follows:

"- Maternal Protein Restriction (MPR) shifts renal proteome to mitochondrial/redox metabolism.

- MPR causes renal histopathological lesions during aging.

- The impacts of MPR are reflected in cellular and molecular alterations indicative of potential renal dysfunction.

- Nephron-specific and epigenetic alterations indicate targeted functional disruption.

- Persistent changes link early malnutrition to renal and metabolic disease risk."

3. It is unclear at times when the authors are referring to the fetal kidney or adult kidney, such as in the abstract (Lines 44-46) and introduction (Lines 93-96). Please clarify.

(14) 3880 - 0481
l.justulin@unesp.br
R. Prof. Doutor Antonio Celso Wagner Zanin, 250
CEP: 18618-689 - Botucatu/SP, Brazil

Answer: We thank you for the suggestion. We have clarified these parameters in our revised abstract and introduction, as follows:

Abstract:

"…associated with reduced nephron number early in life, in addition to renal dysfunction and hypertension during adult life."

Introduction:

"Gestational MPR is associated with low birth weight, a 28% reduction in nephron number, and impaired renal salt excretion early in life, in addition to a higher risk of chronic kidney failure and hypertension in adulthood…"

4. For transparency, the abstract should state what species of rat and the severity of MPR utilised in this study (global, moderate or severe, state percentage reduction).

Answer: We appreciate the reviewer's excellent suggestion. We have added this information to the abstract of the revised manuscript, as follows:

"…This study investigated the effects of gestational and lactational severe MPR (6% compared to 17% in CTR) on the renal proteome of aged male Sprague-Dawley rats (postnatal day 540) using integrated histological, molecular, and in silico approaches…"

5. Whilst I commend the authors for their meticulous reporting of their molecular methods. The methods section is dense. This is sometimes attributed to the authors defining terms that aren't critical to the reproducibility of the experiment; for example, they define terms such as rotary microtome (line 153), H&E (line 156), and mast cell (line 163). Please consider cutting unnecessary words to streamline the information and improve readability.

Answer: We appreciate the reviewer's excellent point. Indeed, some definitions were excessive, redundant, and unnecessary. We have therefore removed this unnecessary information and made our methods section more concise.

(14) 3880 - 0481
l.justulin@unesp.br

R. Prof. Doutor Antonio Celso Wagner Zanin, 250
CEP: 18618-689 - Botucatu/SP, Brazil

6. The authors state that the animals received gestational and lactational MPR; however, offspring are raised until postnatal day 540. Did the pups return to the control diet post-weaning? This should be stated.

Answer: We thank you again for this excellent point. After weaning, both the CTR and GLLP animals received a normoprotein diet until postnatal day 540. We have clarified this information in the revised manuscript, as follows:

"After weaning, animals from both the CTR and GLLP groups received a normoprotein diet until postnatal day (PND) 540. At PND540, male offspring were euthanized by an overdose of ketamine and xylazine, followed by decapitation…"

7. Line 143: Where was the blood collected from?

Answer: Thank you for the question. Following decapitation, the blood was collected directly, then centrifuged and stored (frozen) for future biochemical analyses. We clarify this information in the revised manuscript:

"…After decapitation, blood was collected and centrifuged, and the resulting serum was used for biochemical analysis…"

8. The authors are transparent that this study exclusively used male offspring for molecular analysis. What was the fate of female offspring? It is unclear if they were also euthanised. This should be made clear.

Answer: Thank you very much for the question. In fact, the females were weaned alongside the males on PND21, but some of them were used for other analyses—specifically reproductive ones—by colleagues in the group. We acknowledge that the lack of female analyses is an important limitation and will be addressed in our future studies. Our previous work primarily focused on reproductive and prostatic outcomes (PMID: 37972683; PMID: 33049715; PMID: 37907720; PMID: 39639045; PMID: 36499183), before expanding to systemic and metabolic mechanisms in male offspring (PMID: 38880278; PMID: 39986598; PMID: 40032160; PMID: 42025250; PMID: 42091765; PMID: 41106765; PMID: 39218056). More recently, we have initiated studies in female offspring, investigating the liver, cardiovascular system, and

(14) 3880 - 0481
l.justulin@unesp.br

R. Prof. Doutor Antonio Celso Wagner Zanin, 250
CEP: 18618-689 - Botucatu/SP, Brazil

hypothalamic-pituitary-thyroid-adrenal axis (PMID: 42402324; PMID: 41407027; PMID: 40300334). These ongoing investigations are expected to clarify the sex-specific effects, and we intend to investigate, in future studies, the female renal effects of MPR.

We have highlighted this in the revised manuscript.

"…The females underwent the same treatment and, after weaning, were assigned to other colleagues for reproductive analyses. Here, we used only the male offspring, as noted in the study limitations…"

9. The statistical analysis section states that data is expressed as mean {plus minus} standard deviation. However, the Table 1 legend states that the data are expressed as mean {plus minus} standard error.

Answer: Thank you for the comment. We have added the correct information to section 2.6, as follows:

"…Data were expressed as mean± standard deviation (SD) or Standard Error of the Mean (SEM)…"

10. Table 1 should present all P values for transparency. Consider adding a 4th column to display P values.

Answer: Thank you for the suggestion. We added p-values in Table 2 of the revised manuscript:

*"**Table 2.** Biometric and metabolic parameters in the offspring at PND540.*

Parameters	***CTR***	***GLLP***	
Body mass (g)	*537.9±126.5*	*513.3±61.3*	*p=0.5380*
Kidney mass (g)	*4.121±0.6*	*3.726±0.6*	*p=0.1205*
AST (U/L)	*102.0±30.1*	*105.0±61.7*	*p=0.9037*
ALT (U/L)	*6.7±1.5*	*10.0±3.6*	*p=0.0620*
Creatinine (mg/dL)	*0.59±0.09*	*0.76±0.13**	*p=0.0337*
Alkaline phosphatase (U/L)	*281.6±77.69*	*391.0±101.0*	*p=0.0649*
Urea (mg/dL)	*22.53±3.672*	*23.62±1.796*	*p=0.2403*

**Statistically significant difference between control (CTR) and gestational and lactational protein restriction (GLLP). Data are presented as mean ± SEM (n=6/group). Statistical analysis by the "t" test,*

(14) 3880 - 0481
l.justulin@unesp.br
R. Prof. Doutor Antonio Celso Wagner Zanin, 250
CEP: 18618-689 - Botucatu/SP, Brazil

with significance when p-value < 0.05."

11. Figure 1 demonstrates the effort made by the authors to understand the histology of these kidneys. However, the graphs are small and hard to see relative to the representative histology images.

Answer: We appreciate the comment. We have enlarged the graphs in Figure 1 to improve visibility. The modified Figure 1 is shown below:

(14) 3880 - 0481
l.justulin@unesp.br

R. Prof. Doutor Antonio Celso Wagner Zanin, 250
CEP: 18618-689 - Botucatu/SP, Brazil

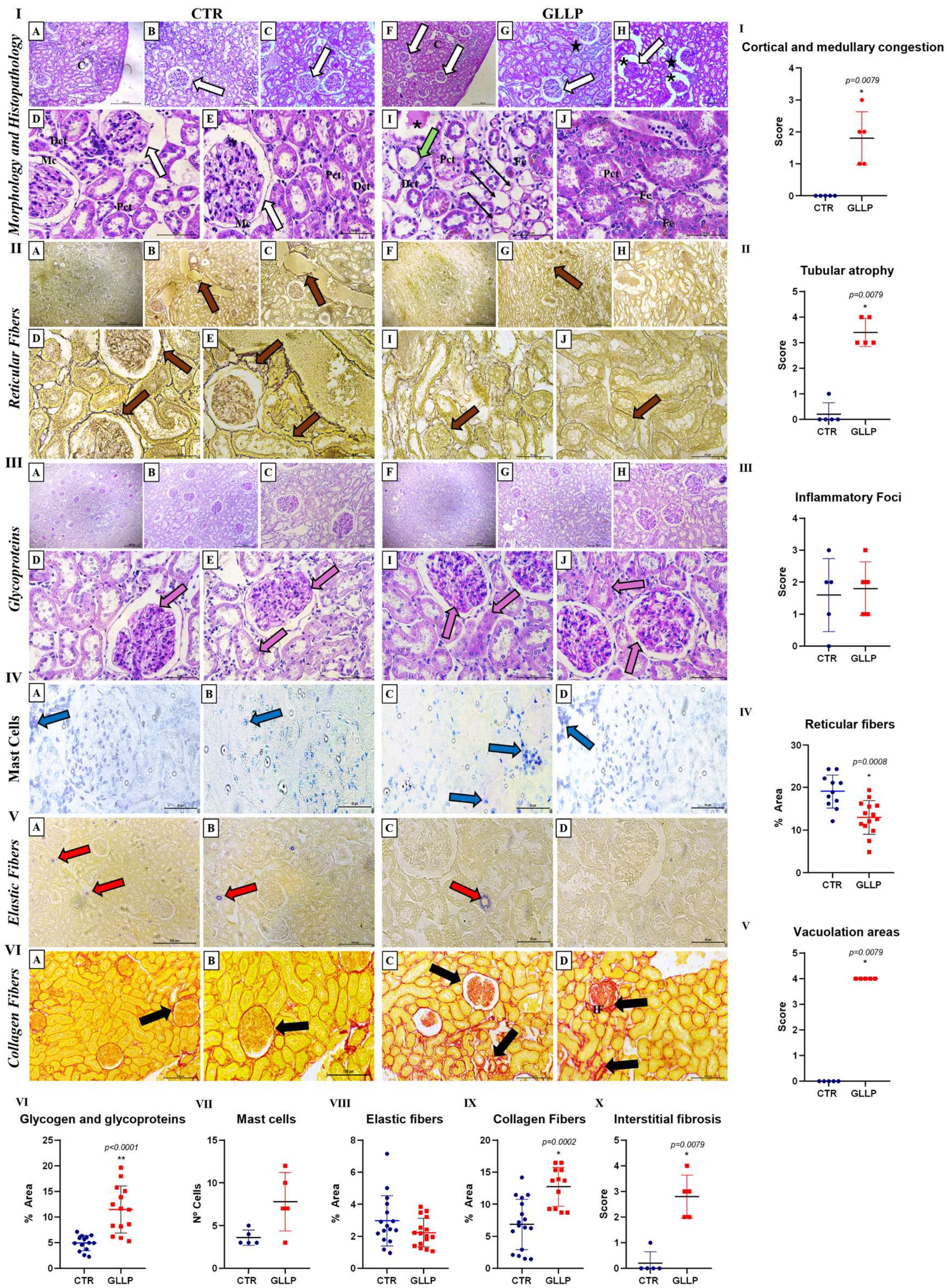

unesp UNIVERSIDADE ESTADUAL PAULISTA
"JÚLIO DE MESQUITA FILHO"
Câmpus de Botucatu

(14) 3880 - 0481
l.justulin@unesp.br

R. Prof. Doutor Antonio Celso Wagner Zanin, 250
CEP: 18618-689 - Botucatu/SP, Brazil

12. The authors have made great attempts to interpret their data in the discussion. Some concepts feel repetitive and could be merged to streamline the message and condense this section (Line 738-41, 840-846, 911-923)

Answer: We appreciate this excellent suggestion. We have consolidated, refined, and summarized these repetitive passages into a single section to make the message more direct and integrative regarding the results, as follows:

"...Our findings demonstrate that the long-term effects of MPR persist into aging, manifesting as metabolic overload, oxidative imbalance, and disrupted transport processes, all of which are accompanied by histopathological alterations and may increase susceptibility to renal disease. Proteomic analyses indicate upregulation of pathways related to acetylation, redox homeostasis, energy metabolism, and mitochondrial function, whereas processes involved in cellular regulation, cytoskeleton-dependent transport, secretion, and muscle contraction are downregulated. These alterations suggest a chronic stress state associated with structural renal damage, metabolic dysregulation, and endocrine-dependent mechanisms. Nephron-segment mapping further indicates that MPR predominantly affects the glomerulus, proximal convoluted tubule, distal convoluted tubule, and collecting duct, supporting impairments in glomerular filtration, tubular metabolic activity, and hormone-dependent renal function. Finally, MPR appears to promote both aerobic and anaerobic metabolic overload, accompanied by activation of antioxidant, protein repair, and cellular stress pathways, highlighting persistent metabolic and redox adaptations in the aging kidney."

13. The authors acknowledge that the use of only males is a limitation, as the responses reported may be sex-specific. In addition, the authors should provide a brief justification for the exclusive use of males. Furthermore, what phenotypes might the authors have expected in female adult kidneys? The literature shows that hypertension in offspring born to poor in utero environments can be male-predominant.

Answer: Thank you very much for the comment. We have reinforced and revised the section of our discussion addressing limitations to highlight these points—including our hypothesis regarding females—as follows:

(14) 3880 - 0481
l.justulin@unesp.br
R. Prof. Doutor Antonio Celso Wagner Zanin, 250
CEP: 18618-689 - Botucatu/SP, Brazil

"This study has several limitations. First, we did not perform direct functional assessments of renal physiology, such as glomerular filtration rate, tubular transport, renal clearance, or urinary biomarkers, limiting functional confirmation of the observed alterations. Second, as this was a proteomics-driven study, mechanistic validation relied primarily on in silico analyses, and further molecular and functional validation is warranted. Third, our analyses were restricted to male offspring. This choice was based on previous evidence demonstrating that maternal protein restriction preferentially reduces nephron number and predisposes males to renal dysfunction and hypertension. Nevertheless, the absence of female data remains an important limitation. Although females are also expected to be affected, sex-specific developmental and epigenetic mechanisms, together with the protective effects of female sex hormones, may differentially modulate renal programming. Future studies should therefore investigate these sex-dependent responses. Finally, although the rat is a well-established model for developmental programming, species-specific differences should be considered when translating these findings to humans. In addition, only maternal protein restriction was evaluated; future studies should examine other nutritional insults, including caloric restriction, high-fat diets, and postnatal environmental adversities. Despite these limitations, our findings provide a strong foundation for understanding the developmental origins of renal dysfunction induced by maternal malnutrition and for guiding future mechanistic, translational, and clinical studies."

14. In addition to my above comments, authors should be cautious not to generalise findings as it is not appropriate to directly compare the adult data to both male and female adult kidneys. Where possible, when referencing outcomes from other studies, state whether the previous findings were sex-specific or in both sexes (Lines 670-680, 699, 719-736, 769).

Answer: Thank you very much. We would like to highlight that the studies described in the discussion involved only males—a point that also underscores the gap in the literature regarding studies on females. As follows:

"Also, during fetal life, other authors observe that MPR (9%) causes adverse effects on sodium regulation mechanisms and response to angiotensin in male rats (Alwasel et al., 2010; Torreggiani et al., 2020)…"

(14) 3880 - 0481
l.justulin@unesp.br
R. Prof. Doutor Antonio Celso Wagner Zanin, 250
CEP: 18618-689 - Botucatu/SP, Brazil

"…The most interesting finding is that the literature shows male renal alterations in response to MPR, similar to those seen in our analyses, but mainly in fetal and adult life, highlighting the potential long-term effects. For example, Gomes et al. (2023) observed that MPR reduced nephron number in male fetus, a condition related to changes in the HIF-1α signaling pathway, NOS, Ep300, and HSP90 expression. Male rats exposed to MPR (10%) during pregnancy and lactation demonstrated reduction in the number of nephrons and renal blood flow, as well as vasoactive, antioxidant, and anti-inflammatory molecules, although they showed resilience to acute kidney injury during adult life (García-Ortuño et al., 2019). Guo et al. (2020) showed that MPR modulates the overall proteomic profile of fetal kidneys, enriching biological pathways associated with ion channels/transporters, reducing the expression of aquaporins 2/4, causing an imbalance in solute transporter isoforms (SLCs), as well as increasing the expression of pathways associated with apoptosis and increasing ATP6V1C2 – some molecules also found in our model, with patterns of expression differences. An experimental study evaluated the effects of gestational and lactational MPR or neonatal overnutrition on young male animals, and observed increased lipid peroxidation, as well as reduced glutathione, total thiol content, and renal superoxide dismutase and catalase activities (Pedroza et al., 2019). These data are quite interesting as they reveal disturbances in the antioxidant system and metabolic aspects in the kidneys of animals exposed to MPR, but still in the early stages of life."

"From a DOHaD perspective, Alvarez et al. (2026) demonstrate that MPR induces early-life renal impairment in male rats, accompanied by widespread proteomic reprogramming…"

15. The authors could justify the use of a 6% protein restriction, which is considered severe. Does the severity of MPR reflect that experienced globally in women? Discussion on how this might compare to a moderate MPR or a diet restricted in more substrates (global) may be useful.

Answer: We thank the reviewer for this important comment. We agree that a 6% protein diet represents a relatively severe degree of maternal protein restriction and does not necessarily reflect the full spectrum of maternal malnutrition observed worldwide. However, this model has been extensively validated and widely used in DOHaD research, as it consistently induces fetal programming while maintaining maternal viability and pregnancy success, thereby enabling

(14) 3880 - 0481
l.justulin@unesp.br

R. Prof. Doutor Antonio Celso Wagner Zanin, 250
CEP: 18618-689 - Botucatu/SP, Brazil

mechanistic investigation into the long-term consequences of protein deficiency. Furthermore, it is worth noting that contexts of food insecurity and hunger are reflected, at least in part, by protein deficits; however, future translational and clinical studies should certainly examine these contexts of maternal malnutrition and their renal effects through the lens of DOHaD.

"From a translational perspective, our severe MPR (6% protein) model represents an isolated reduction in protein intake that may resemble conditions of severe food insecurity or famine; however, it does not encompass the full complexity of human malnutrition. In clinical settings, maternal malnutrition is highly heterogeneous and commonly involves moderate protein deficiency combined with energy and micronutrient deficiencies. Therefore, future clinical and epidemiological studies should investigate the real human nutritional insults within the DOHaD framework to better define their effects on embryo-fetal programming and the long-term risk of renal disease."

END OF COMMENTS

unesp UNIVERSIDADE ESTADUAL PAULISTA
"JÚLIO DE MESQUITA FILHO"
Câmpus de Botucatu

(14) 3880 - 0481
l.justulin@unesp.br

R. Prof. Doutor Antonio Celso Wagner Zanin, 250
CEP: 18618-689 - Botucatu/SP, Brazil

Dear Dr Justulin,

Re: JP-RP-2026-291758R1 "**Maternal Protein Restriction Programs the Aging Kidney: Proteomic Signatures of Early-Life Origin Renal and Metabolic Dysfunction**" by Marina Pereira Pires, Matheus Naia Fioretto, Marcel Rodrigues Ferreira, Isabelle Tenori Ribeiro, Luisa Annibal Barata, Flávia Alessandra Maciel, Lucas Sobrinho Lemos, Renato Mattos, Luiz Marcos Frediani Portela, Patrick Vieira Souza, Luisa Correia Gomes, Gustavo Monezzi Cordeiro, Wellerson Rodrigo Scarano, Patricia Aline Boer, Jose Antonio Rocha Gontijo, and Luis Antonio Justulin

We are pleased to tell you that your paper has been accepted for publication in The Journal of Physiology.

The last Word (or similar) version of the manuscript provided will be used by the Production Editor to prepare your proof. When this is ready you will receive an email containing a link to Wiley's Online Proofing System. The proof should be thoroughly checked and corrected as promptly as possible.

Authors should note that it is too late at this point to offer corrections prior to proofreading (***please confirm correct spelling of author: Luiz Marcos Frediane Portela - or is 'Frediani' the correct spelling?***). Major corrections at proof stage, such as changes to figures, will be referred to the Editors for approval before they can be incorporated. Only minor changes, such as to style and consistency, should be made at proof stage. Changes that need to be made after proof stage will usually require a formal correction notice.

All queries at proof stage should be sent to: TJP@wiley.com.

Following copyediting and typesetting the accepted version will be published online.

Yours sincerely,

Kim Barrett
Senior Editor
The Journal of Physiology

IMPORTANT POINTS TO NOTE FOLLOWING ACCEPTANCE OF YOUR PAPER:

• IMPORTANT NOTICE ABOUT OPEN ACCESS: To assist authors whose funding agencies mandate immediate public access to published research findings, The Journal of Physiology allows authors to pay an Open Access (OA) fee to have their papers made freely available immediately on publication.

The Corresponding Author will receive an email from Wiley with details on how to register or log in to Wiley Authors where you will be able to place an order.

• You can check if your funder or institution has a Wiley Open Access Account here:
https://authors.wiley.com/author-resources/Journal-Authors/open-access/author-compliance-tool.html

MEMBER DISCOUNT: Corresponding authors who are Members of the Physiological Society are eligible for a 20% discount on the cost of the Article Processing Charge (APC). To claim this discount, the Corresponding Author will need to log in to the Physiological Society Membership portal and retrieve the discount code (https://www.physoc.org/journals-and-media/journals/journal-discount-for-members/). This code should then be quoted in the billing section of Wiley Authors when selecting the open access option.

• TRANSPARENT PEER REVIEW POLICY: To improve the transparency of its peer review process, The Journal of Physiology publishes online as supporting information the peer review history of all articles accepted for publication. Readers will have access to decision letters, including Editors' comments and referee reports, for each version of the manuscript as well as any author responses to peer review comments. Referees can decide whether or not they wish to be named on the peer review history document.

• You can help your research get the attention it deserves! Check out Wiley's free Promotion Guide for best-practice recommendations for promoting your work at: www.wileyauthors.com/eeo/guide. You can learn more about Wiley Editing Services which offers professional video, design, and writing services to create shareable video abstracts, infographics, conference posters, lay summaries, and research news stories for your research at: www.wileyauthors.com/eeo/promotion.

• If you would like to receive our 'Research Roundup', a monthly newsletter highlighting the cutting-edge research published in The Physiological Society's family of journals (The Journal of Physiology, Experimental Physiology, Physiological Reports,

The Journal of Nutritional Physiology and The Journal of Precision Medicine: Health and Disease), please click this link, fill in your name and email address and select 'Research Roundup':
https://www.physoc.org/journals-and-media/membernews

EDITOR COMMENTS

Reviewing Editor:

Comments to the Author (Required):
Thank you for addressing the reviewers comments and revising the paper.

REFEREE COMMENTS

Referee #1:

The authors have adequately responded to all of my earlier questions and comments. The manuscript is now much improved.

Referee #2:

Thank you for addressing the comments raised in detail. The manuscript is much improved. Overall, this work makes an impactful contribution to the field. Well done.
